# Native Seed Supply and the Restoration Species Pool

**DOI:** 10.1111/conl.12381

**Published:** 2017-06-19

**Authors:** Emma Ladouceur, Borja Jiménez‐Alfaro, Maria Marin, Marcello De Vitis, Holly Abbandonato, Pietro P.M. Iannetta, Costantino Bonomi, Hugh W. Pritchard

**Affiliations:** ^1^ Botany Section Museo Delle Scienze (Muse) Trento Italy; ^2^ Department of Earth and Environmental Science University of Pavia Pavia Italy; ^3^ The German Centre for Integrative Biodiversity Research (iDiv) Halle‐Jena‐Leipzig, Deutscher Platz 5e 04103 Leipzig Germany; ^4^ Geobotany and Botanical Garden, Institute of Biology Martin Luther University Halle Wittenberg Am Kirchtor 1 06108 Halle (Saale) Germany; ^5^ Scotia Seeds Mavisbank, Farnell, Brechin UK; ^6^ The James Hutton Institute Dundee UK; ^7^ Royal Botanic Gardens, Kew Wellcome Trust Millennium Building Wakehurst Place West Sussex RH17 6TN UK

**Keywords:** Biodiversity, ecological restoration, European grasslands, revegetation, seed germination, seed production

## Abstract

Globally, annual expenditure on ecological restoration of degraded areas for habitat improvement and biodiversity conservation is approximately $18bn. Seed farming of native plant species is crucial to meet restoration goals, but may be stymied by the disconnection of academic research in seed science and the lack of effective policies that regulate native seed production/supply. To illustrate this problem, we identified 1,122 plant species important for European grasslands of conservation concern and found that only 32% have both fundamental seed germination data available and can be purchased as seed. The “*restoration species pool,”* or set of species available in practice, acts as a significant biodiversity selection filter for species use in restoration projects. For improvement, we propose: (1) substantial expansion of research and development on native seed quality, viability, and production; (2) open‐source knowledge transfer between sectors; and (3) creation of supportive policy intended to stimulate demand for biodiverse seed.

## Introduction

One‐tenth of global wilderness has been destroyed in the last two decades (Pennisi 2016), and two‐thirds of terrestrial environments are officially classed as degraded (Merritt & Dixon 2011). Ecological restoration (ER) accelerates the recovery of a degraded ecosystem with respect to health, integrity, and sustainability (SER 2004), and is recognized as a key complementary action for habitat conservation. Current global *ER* targets aim to restore 150 million ha or 15% of degraded ecosystems by 2020 (Menz *et al*. 2013). The estimated $18bn/year restoration cost is far exceeded by the potential global ecosystem service benefits of $85bn annually (Menz *et al*. 2013). Critical to success is the urgent need for access to high‐quality seed through the farming of native species, as part of a range of flexible strategies to improve *ER* (Broadhurst *et al*. 2016).

Several large‐scale *ER* initiatives are underway globally, such as the Australian Gondwana Link (Merritt & Dixon 2011), the Bureau of Land Management U.S. initiatives (Oldfield & Olwell 2015), the African Great Green Wall (Sacande & Berrahmouni 2016), and the European Union (EU) Natura 2000 (European Commission 1992). Seed‐based plant conservation and use strategies (Merritt & Dixon 2011; Royal Botanic Gardens Kew 2015), seed‐based research (Jiménez‐Alfaro *et al*. 2016), and seed supply all play critical roles in successful ER. However, native seed sourcing, collection, production, and storage is more challenging than for agricultural species (Bischoff *et al*. 2008; Broadhurst *et al*. 2008) for which cultivars have been bred to be stable, uniform, and distinct (European Commission 1966).

*ER* depends on selecting appropriate species to cope with abiotic and biotic characteristics of degraded habitats. In ecological communities, scientists describe the species pool as the set of species that potentially occur at a site (Zobel 1992). The conditions limiting or facilitating species assembly will determine successional and recovering legacies of a system, including responses following *ER* (Temperton *et al*. 2004). Hand‐collecting seed in large quantities from a broad range of species is unrealistic for most *ER* projects and wild populations risk depletion. Often the material used is restricted to that available from commercial or institutional seed suppliers. The “*restoration species pool*” (“*RSP”*), or pool of species available from these seed suppliers, thus imposes a critical biodiversity filter in *ER* projects. Where native supply lacks, easily available agronomic or horticultural seeds are used as a substitute, which is ecologically unacceptable. A *RSP* of native species, which has been systematically sourced between and within populations and species distribution ranges, is necessary for the support of genetic diversity in seed supplies and restored ecosystems (Hoban & Schlarbaum 2014).

Seed yields and germination of wild species can be naturally low and variable (Fenner 2000), and while cropping of native species can facilitate controlled production, some seed ecological traits (Fenner & Thompson 2005) can determine obstacles to harvesting. Not all wild species are candidates for commercial production as variation in seed morphological traits necessitates the use of appropriate harvesting and conditioning equipment, the costs of which can be very high if a large number of species are being produced. Proper seed management from collection to postconditioning storage is essential to maintain seed viability, which is variable between suppliers and can be very low (Marin *et al*. 2017). These challenges require collaborative efforts between seed suppliers and researchers to fully realize the potential of providing native farmed seeds for ER. This encompasses research on seed germination, dormancy (a process that regulates germination so that plants emerge under environmental conditions favourable for seedling establishment; Table S1), seed traits relevant for *ER* (Jiménez‐Alfaro *et al*. 2016), and other bottlenecks that can be encountered such as adaptations for cultivation or genetic diversity maintenance (Chivers *et al*. 2016). However, research findings are rarely accessible to public stakeholders involved in ER.

Here, we assess the potential of the *RSP* to meet conservation needs in European grasslands, which are priority habitats as detailed in European policies on nature conservation. Human‐induced habitat loss has impacted grassland biomes to the greatest rate and extent, largely due to agricultural conversion and the lack of conservation protections (Hoekstra *et al*. 2005). This neglect is in stark contrast to the biodiversity value of temperate grassland habitats, which across continental Europe are global biodiversity hot spots (Wilson *et al*. 2012). Using European grasslands of conservation concern as a case study, we analyze how many species have both detailed seed quality data and commercial seed lots available across taxa and across three species groups of relevance to European policies on *ER*. Addressing the availability of seed and related scientific information is important for the design of effective policy, research agendas, the foci of commercial seed suppliers, and reducing the risk of falling short in reinstating functional ecosystems in *ER* (Menz *et al*. 2013).

## Methods

### Study systems and target species

The European initiative Natura 2000 aims to establish a network of diverse, representative high‐quality protected habitats of conservation concern, much of which will require intensive *ER* (European Commission 1992). Our study is focused on six major temperate grassland habitat types of conservation concern in Europe: lowland meadows (Natura 2000 number: 6510); high altitude hay meadows (6520); dry grasslands (6210); species rich *Nardus* grasslands (6230); calcareous alpine grasslands (6170); and acidic alpine grasslands (6150).

We created a database of 1,122 target species with potential interest for *ER* within these habitats, regulated by EU legislation that affects strategies of seed quality and use (Table S2). This includes 116 *protected* species subjected to legal protection, in most cases endangered or narrow endemic species; 929 *indicator* species, which are indirectly protected when occurring in protected habitats but unregulated in seed production; and 77 *fodder* species controlled for quality as domestic stock feed (European Commission 1966; 2014), as well as for preservation of genetic diversity (European Commission 2010; Table 1).

**Table 1:** Relevant legislation details related to each target species group

Species group	Description	Legislation	Impact
Protected species (*N* = 116)	Includes species of conservation concern, in most cases endangered or narrow endemics, listed by name in relevant policy, and occurring in focus habitats.	Specific species for which member states must protect and conserve when found to occur under Annex II & IV of the EU policy on Conservation of Natural Habitats Wild Fauna and Flora (European Commission 1992).	Species seed cannot be collected without a rigorous permit process.
Indicator species (*N* = 929)	Species that are diagnostic or dominant for any of the selected habitats at the continental scale according to Schaminée *et al*. (2016) and vegetation ecology literature (Georg Grabherr & Mucina 1993).	These species are indirectly conserved in Annex II as reflected in the designation of special protected areas for the habitats in that they occur under the EU policy on Conservation of Natural Habitats Wild Fauna and Flora (European Commission 1992).	Species are of interest for use in restoration and have no direct EU policy restrictions on their collection, reproduction, or use but may have local regulations.
Fodder species (*N* = 77)	Grass and legume species used for animal forage, also considered valuable for preservation of the natural environment and conservation of genetic resources in grasslands listed by name under relevant policies.	Specific species and genera important for domestic stock and grazing (European Commission 1966, 2010, 2014).	Controlled for quality including high purity standards and minimum germination thresholds in EU Commission Directive 1966. Expanded in Directive (2010) to include harvest method, seed weight, quantity, region of origin, source area (collection site and multiplication), habitat type, and year of collection. Native seed production cannot exceed 5% of the total commercial cultivar production market in their country.

*N* = number of species in each group.

To assess the availability of seed quality data, we collected trait information on germination temperature and dormancy type of the target species available from the *Seed Information Database* (Royal Botanic Gardens Kew 2008), and the most recent review of seed germination studies (Baskin & Baskin 2014). As these are the main traits related to the germinability of a seed lot, we assume that having this information implies a minimum contribution of the scientific community for a given species. A systematic online search was conducted from November 2014 to May 2016, and the lists of species available commercially as seed were downloaded, or requested to seed suppliers. As there are multiple seed sources in some countries, the supplier providing the highest number of target species was selected since the inclusion of smaller companies did not influence the total number of available species. This resulted in seed availability lists from 17 seed suppliers across 17 countries (Table S3). Species names were verified against *The Plant List* (Missouri Botanical Gardens, Royal Botanic Gardens Kew 2013). Possible limitations of these data are that species reported as available may be an overestimate as lists may be outdated, inaccurate, or in some cases represent cultivars rather than native species, particularly in the *fodder* group. Nonetheless, the list is an accurate representation of the current state of native seed acquisition in Europe. We use the term *supplier* instead of *producer* because in the majority of cases, seed is reproduced in a native seed farm or orchard, but in some cases seed may be hand‐collected.

### Analyses

Data were collected as binomial variables. To assess *Germination Data Availability (GDA)*, each species was assigned as data being available (1) or not (0). Similarly, species were either *Commercially Available (CA)* (1) or not (0).

The proportions (%) of species with *CA* and with *GDA* were calculated for each plant family represented in the target species list to elucidate taxonomic representation as a surrogate of phylogenetic variation. A Generalized Linear Model (*GLM*) was fitted to assess the variation of *CA* as a function of *GDA* and species groups. The *GLM* was computed with binomial error distribution and logit link function in order to assess the influence of policy groups and *GDA* (explanatory variables) on *CA* (response variable; *CA* ∼ *GDA* + policy group). All analyses were performed in *R Statistical Computing Language and Platform version 3.2.2* (R Core Development Team 2016), and Figures created in the package *ggplot2* (Wickham 2009) and *yarrr* (Phillips 2016). The package *Effects* (Fox 2003) was used to create probability estimates of *CA* based on each variable and package *PMCMR* for post hoc pairwise Kruskal‐Wallis tests (Pohlert 2014).

## Results

**Figure 1:**
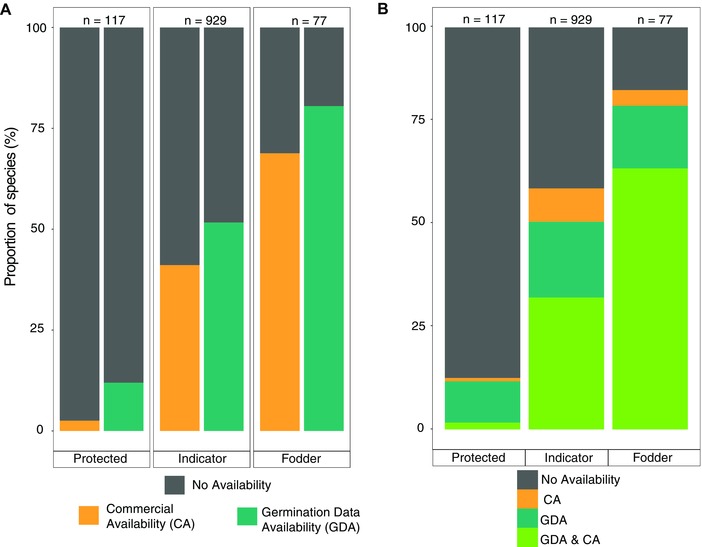
(A) Proportion (%) of species that are commercially available (*CA*) and with germination data availability (*GDA*) (B) proportion (%) of species that are commercially available (*CA*) with germination data availability (*GDA*), and with the combination of *CA* + *GDA*. *N*: number of species represented within each group.

The 1,122 target species with potential interest for *ER* within European grassland habitats are spread across 59 plant families, with highest representation in Compositae (146 species) and with the top 5 and 10 families comprising 43% and 62% of the species list, respectively. Information on *GDA* and *CA* alone extended to 49% (i.e., 556) and 39% (i.e., 439) of target species, respectively (Figure 1A). Information for both seed *GDA* and *CA* details are available for only 32% (i.e., 358) of species on the target list (Figure 1B). Supplied seed is not available across all suppliers (Figure S1), although *indicator a*nd *fodder* species with *GDA* are available across a higher proportion of suppliers than those without *GDA* and with *protected* status (Kruskal‐Wallis s^2^ = 338.81, *P* ≤ 0.001; Tables S2 and S4).

**Figure 2:**
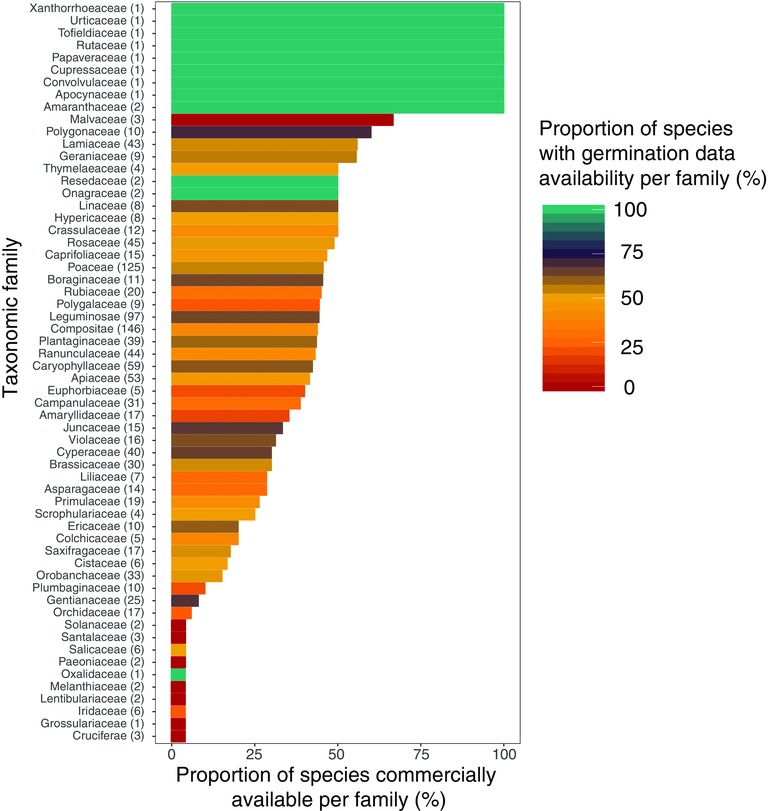
Bars show the proportion (%) of species per taxonomic family that have seed which has *commercial availability*. The degree and proportion of *germination data availability* is represented by the color scale according to the Seed Information Database (*Royal Botanic Gardens*, Kew 2008) and Baskin & Baskin (2014). The numbers in brackets next to each family name represents how many species are included in the data set from that given family.

The majority of taxonomic families completely lacking *GDA* are also completely lacking *CA*, although the sample size is small in these cases (Figure 2, Table S5). The vast majority of families with large sample sizes have ∼50% *GDA* and *CA*. Within this case study, there are seven families, spanning nine genera and 15 species, for which germination data are unknown. Twelve families (20% of total) lie within the lower quartile of *CA*, covering 158 species (14% of total).

**Figure 3:**
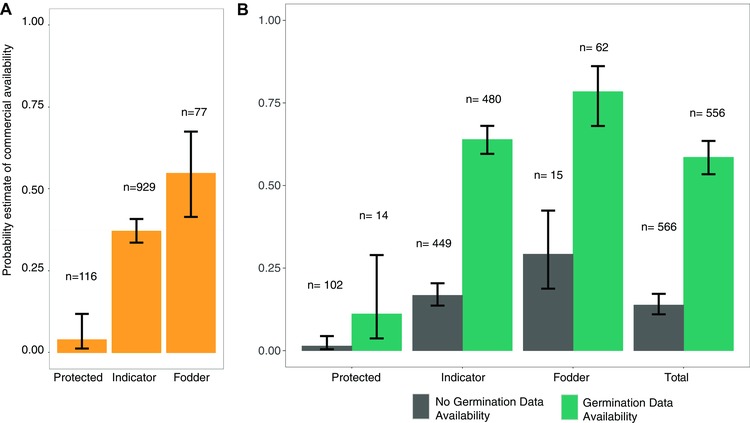
Predicted effect plots showing the *commercial availability* of species grouped per species category. Probability was estimated using *GLM* (binomial error, logit link) fitted to the commercial production data of each species (*commercial availability* ∼ *germination data availability* + species group). The same model was used to fit each group, and results were grouped based on: (A) species groups (*protected, indicator, fodder*) (B) species group + *germination data availability*. Bars represent the probability that a given group of species is commercially available. Brackets represent the upper and lower limits of that estimate. *N* = number of species represented by each prediction.

Strong predictive patterns based on the *GLM* are exhibited for the estimate of *CA* of target species across all variables (Figure 3, Table 2). The model predicts that *protected* species have a 0.04 probability of being *CA*, *indicator* species 0.37 (*P* < 0.001), and *fodder* species 0.54 (*P* < 0.001; Figure 3A, Table 2). Species with no *GDA* have 0.13 probability of being *CA*, and species with *GDA* have a 0.58 probability of being *CA* overall (*P* < 0.001; Figure 3B). The combination of predictors (Figure 3B) provides a further level of outcomes. *Protected* species for which there is no *GDA* have 0.01 probability of *CA*; this probability increases to 0.11 when there is *GDA*. Comparable values for *indicator* species without and with *GDA* are 0.17 and 0.64, respectively; and 0.29 and 0.78 probability, respectively, for *fodder* species.

**Table 2:** Generalized linear model (binomial error, logit link) analysis testing the effect of *germination data availability* and species group on *commercial availability*

Coefficient	Effect estimate	Standard error	Z	*P*
Intercept (protected)	−4.2541	0.6016	−7.071	< 0.001
Germination data availability	2.1759	0.1524	14.281	< 0.001
Indicator species	3.3685	0.6570	5.127	< 0.001
Fodder species	2.6502	0.6021	4.401	< 0.001

Results were estimated using *GLM* fitted to the commercial availability data for each species.

## Discussion

### The *Restoration Species Pool in European grasslands*

To our knowledge, no studies have investigated the availability of commercial seed and related germination data for native seed in a large‐scale case study. In Europe, the relatively high availability of native seeds for *fodder* species demonstrates that commercial availability of native seed is subject to economic demand and a long‐standing regulatory framework. This framework follows an agricultural model meant for animal feed rather than Ecological Restoration (ER) (European Commission 1966), yet is recognized for *ER* use (European Commission 2010). The opposite trend is evident for *protected* species, as the availability of commercial and germination data is extremely low, despite their conservation concern in EU regulations. There are relatively high levels of *indicator* species represented in the Restoration Species Pool (RSP), but this does not necessarily indicate availability from many suppliers. Seed availability and use is compounded by the origin of the seed, as some supplies may not be appropriate for use in every region (Bischoff *et al*. 2008). Species for which there is a lack of *GDA* are also less likely to be *CA* and more likely to be omitted from the *RSP*. This indicates the urgent need for research and development on European grassland native seed biology, including knowledge transfer to support the commercial sector.

When there are little or no germination data for species within a family, congeneric species can offer predictions of potential dormancy (Table S1), that is, *implied dormancy*, and thus the type of environmental conditions to trigger germination (Baskin & Baskin 2014). Implied dormancy for the large majority of study species (∼75%) indicates probable complex germination characteristics (Table S1). Currently, most revegetation projects in Europe have no requirement to improve biodiversity outputs, thus there is lack of consistent demand, and little capacity to improve the range of species with *CA*, particularly for species that may be complex to supply. Without change, *ER* of grassland habitats could continue to demonstrate species bias limiting biodiversity, facilitating the persistence of degraded systems in alternatively stable states (Suding *et al*. 2004). Improving the *RSP* will reduce risk in *ER* projects as a complementary conservation resource.

For the *RSP* to better support *ER*, industry also requires cooperative market sharing, improved provision and storage strategies. In Australia, United Kingdom, and the United States, there are examples of government, community, or nonprofit groups working cooperatively with seed suppliers to enable the inclusion of species that have challenging seed traits in the commercial *RSP* supply chain. The U.S. Native Plant Program (Oldfield & Olwell 2015) contracts production of seed across all available suppliers, to partition demand and market share, then stored in government infrastructure for purchase. As a unique example in Europe, Germany has mandated that only native species may be used for all revegetation by 2020 (BNatSchG, Federal Ministry for the Environment, Nature Conservation, and Nuclear Safety 2010). Compared to German native seed demand in 2015, production of local native seeds must grow 10‐fold to meet 2020 targets (Pers. Comm., Ann Kareen Mainz, Association of German Wild Seed Producers), an increase which will require expansion of their *RSP*. Demand creation, contracting, storage, and provision solutions must be developed in tandem to effectively expand *RSP* capacity.

### Policy recommendations

Current legislation relating to *protected* (European Commission 1992) and *fodder* (European Commission 2010) species recognize the need to produce seed specifically for ER, but do not match the native seed market appropriately. Policy relating to the use of seed mixtures mandates that commercially produced seed must come from the same source area in which it is being used, and germination minimums are required (European Commission 2010), which are easily achievable in cultivars, but unrealistic for native species. These quality standards are too restrictive (Tishew *et al*. 2011), to which there is low adherence and enforcement, as they are contradictory to a much‐needed industry with a small market niche. Supportive regulation is needed and future EU policy should require that all public revegetation projects use only native material. Creating demand through policy while aligning the contracting of supply offers immense potential to enable growth of the *RSP*. We strongly support initiation of policies to contract annual native seed production of baseline *indicator* and *fodder* species across available producers to store for large‐scale projects. Policy should require vegetation biodiversity targets to be met in *ER* and revegetation. Sourcing and contracting of site‐specific seed material beyond yearly *indicator* and *fodder* stores (including but not limited to *protected* species) should be required at project inception to allow time for realistic production. New policies should be designed to embrace consultation with the native seed industry and restoration professionals.

Conservation seed banks for native species can support these strategies in a small capacity and can provide access to relevant small‐scale seed processing and quality assessment equipment (Nevill *et al*. 2016). The largest *ex situ* plant conservation programme globally, the *Millennium Seed Bank Partnership (MSBP*), managed by the Royal Botanic Gardens, Kew, UK, has successfully banked seeds of 13% of the world's wild species, aiming to bank 25% by the year 2020 (Royal Botanic Gardens Kew 2015). Seed from the *MSBP* has been used for small‐scaled re‐establishment, generally targeted for threatened species. An exemplar is FAO‐RBG Kew “Africa's Great Green Wall” program within which collaborating country seed banks supply ∼25,000 kg of seed per annum of about 200 species of trees, shrubs, and grasses (Sacande & Berrahmouni 2016). Nevertheless, a new form of *Restoration Seed Bank*s (Merritt & Dixon 2011) is needed if a sustainable seed supply chain of the right scale is to be supported for the *RSP*. To improve *ER* outcomes, wide expansion of current capacity and collaboration across sectors is needed to provide the requisite tons of native seed needed (Merritt & Dixon 2011). In addition, research in seed biology and vegetation science applied to seed sourcing, applications, and bottlenecks related to collection and use are required.

Current research in seed biology and regeneration processes remains specialized, in need of urgent expansion (Larson & Funk 2016). In addition, long‐term interdisciplinary and collaborative open‐source knowledge sharing platforms are needed to facilitate the exchange of research (Royal Botanic Gardens Kew 2015). We suggest future germination research focus on the development of efficient dormancy breaking treatments, the thermal control of germination (thresholds and rates), and improvements in native seed production practices for European grassland species not currently covered by the *RSP*. Integration of research and industry knowledge sharing where any research project connected to native seed germination delivers findings to the private sector could hold wide benefits. Research projects for *protected* or underrepresented taxa could ideally include commercial or cooperative seed production contracts for direct use in conservation and reintroduction as industry output components. Supplying *protected* species must be strictly designed, implemented, and controlled with the direct use of vanguard science through extremely collaborative approaches (Shirey *et al*. 2013).

## Conclusions

Our analysis presents the first study investigating seed germination data availability and the commercial “*RSP.”* We present a continental case study, reflecting a global issue of global importance to habitat conservation. In summary, we encourage further exploration and reconsideration of public policy, compilation of open‐access knowledge sharing across sectors and multinational efforts to provide infrastructure and support, so as to expand and realise the full potential of the emerging native seed industry. Improving the breadth of seed biology research and knowledge sharing between sectors has potential to support the expansion of the commercial native seed market and the *RSP*. Improved commercial availability could reduce species bias and risk in ER.

## Supporting information


**Figure S1** Observed percentage (%) of suppliers (total 17) with *commercial availability* of seed with and without *germination data availability*.
RDI plots (raw data, descriptive and inference statistics) show jittered points of raw data, center bars indicate the mean of the data, beans outline the smoothed density of the data, whiskers mark the 10% and 90% quantiles of the data, and inference bands show the Bayesian 95% high‐density interval inferential statistics for each group. Letters show statistical differences between groups (Table S4).

**Table S1** Simplified seed dormancy types (adapted from Baskin & Baskin 2014)
**Table S2** Full species list, associated category, and associated data
*CA* = commercial availability (yes [1], no [0]), *GDA* = germination data availability (yes [1], no [0]).
**Table S3** Seventeen seed suppliers across 17 countries used for data collection
**Table S4** Statistics representing differences between variables in the percentage of suppliers with seed of each species commercially available compared across species groups (Figure S1)
Kruskal‐Wallis *χ* ^2^ test and post hoc pairwise Tukey and Kramer (Nemenyi) *χ* ^2^ test, *P*‐value statistics, indicating significance between group variables. Germination data available = “+*GDA*,” germination data not available = “–*GDA*.”
**Table S5** The complete data set summarized by taxonomic family in descending order of percentage of commercial availability (*CA*)
# = number, % = percentage, Sp. = species, *CA* = commercially availability, *GDA* = germination data availability. Click here for additional data file.
