# Supplementary material for: Native Seed Supply and the Restoration Species Pool
Source: Conserv Lett. 2017 Jun 19;11(2):e12381. doi: 10.1111/conl.12381 (PMC5993272; doi:10.1111/conl.12381)
Supplement: Supplementary file 2 — Table S1 Simplified seed dormancy types (adapted from Baskin & Baskin 2014) Table S2 Full species list, associated category, and associated data CA = commercial availability (yes [1], no [0]), GDA = germination data availability (yes [1], no [0]). Table S3 Seventeen seed suppliers across 17 countries used for data collection Table S4 Statistics representing differences between variables in the percentage of suppliers with seed of each species commercially available compared across species groups (Figure S1) Kruskal‐Wallis χ 2 test and post hoc pairwise Tukey and Kramer (Nemenyi) χ 2 test, P‐value statistics, indicating significance between group variables. Germination data available = “+GDA,” germination data not available = “–GDA.” Table S5 The complete data set summarized by taxonomic family in descending order of percentage of commercial availability (CA) # = number, % = percentage, Sp. = species, CA = commercially availability, GDA = germination data availability. [file CONL-11-na-s001.docx]

**Supporting Information**

**Table S1:** Simplified seed dormancy types adapted from (Baskin & Baskin 2014)**.**

| **Dormancy Type** | **Indications of Germination** | **Treatments Required** |
| --- | --- | --- |
| Non-dormant (ND) | Occurs within a few days to > four (4) weeks | None |
| Physiological Dormancy (PD) | Root emergence requires more than four (4) weeks; shoot emergence can be delayed a further four (4) weeks. | Cold stratification treatments |
| Physical Dormancy (PY) | Seeds do not imbibe water | Scarification |
| Morphophysiological Dormancy (MPD) | Seeds can take four (4) weeks or longer to germinate | Cold, warm, cold+warm, or chemical stratification treatments. |

**Table S2:** Full species list, associated policy category, and associated data. CA=Commercial Availability (Yes (1), No (0)), GDA= Germination Data Availability (Yes (1), No (0)).

| Family | Species | Group | Dormancy | Policy | CA | GDA | % of Producers |
| --- | --- | --- | --- | --- | --- | --- | --- |
| Amaranthaceae | *Chenopodium album* | Indicator | NA | NA | 1 | 1 | 17.65 |
| Amaranthaceae | *Chenopodium bonus-henricus* | Indicator | PD | NA | 1 | 1 | 17.65 |
| Amaryllidaceae | *Allium grosii* | Protected | NA | 92/43/CEE | 0 | 0 | 0.00 |
| Amaryllidaceae | *Allium lusitanicum* | Indicator | NA | NA | 1 | 0 | 5.88 |
| Amaryllidaceae | *Allium oleraceum* | Indicator | NA | NA | 1 | 0 | 11.76 |
| Amaryllidaceae | *Allium schoenoprasum* | Indicator | NA | NA | 1 | 1 | 41.18 |
| Amaryllidaceae | *Allium senescens* | Indicator | NA | NA | 1 | 0 | 5.88 |
| Amaryllidaceae | *Allium sphaerocephalon* | Indicator | NA | NA | 1 | 1 | 11.76 |
| Amaryllidaceae | *Allium vineale* | Indicator | NA | NA | 1 | 0 | 23.53 |
| Amaryllidaceae | *Narcissus asturiensis* | Protected | NA | 92/43/CEE | 0 | 0 | 0.00 |
| Amaryllidaceae | *Narcissus bulbocodium* | Indicator | NA | NA | 0 | 0 | 0.00 |
| Amaryllidaceae | *Narcissus calcicola* | Protected | NA | 92/43/CEE | 0 | 0 | 0.00 |
| Amaryllidaceae | *Narcissus cyclamineus* | Protected | NA | 92/43/CEE | 0 | 0 | 0.00 |
| Amaryllidaceae | *Narcissus dubius* | Protected | NA | 92/43/CEE | 0 | 0 | 0.00 |
| Amaryllidaceae | *Narcissus flavus* | Protected | NA | 92/43/CEE | 0 | 0 | 0.00 |
| Amaryllidaceae | *Narcissus poeticus* | Indicator | NA | NA | 0 | 0 | 0.00 |
| Amaryllidaceae | *Narcissus pseudonarcissus* | Indicator | MPD | NA | 0 | 1 | 0.00 |
| Amaryllidaceae | *Narcissus triandrus* | Protected | NA | 92/43/CEE | 0 | 0 | 0.00 |
| Amaryllidaceae | *Narcissus viridiflorus* | Protected | NA | 92/43/CEE | 0 | 0 | 0.00 |
| Apiaceae | *Aegopodium podagraria* | Indicator | MPD | NA | 1 | 1 | 5.88 |
| Apiaceae | *Angelica heterocarpa* | Protected | NA | 92/43/CEE | 0 | 0 | 0.00 |
| Apiaceae | *Angelica palustris* | Protected | NA | 92/43/CEE | 0 | 0 | 0.00 |
| Apiaceae | *Angelica sylvestris* | Indicator | NA | NA | 1 | 1 | 47.06 |
| Apiaceae | *Anthriscus sylvestris* | Indicator | MPD | NA | 1 | 1 | 41.18 |
| Apiaceae | *Apium repens* | Protected | NA | 92/43/CEE | 0 | 1 | 0.00 |
| Apiaceae | *Astrantia major* | Indicator | NA | NA | 1 | 1 | 17.65 |
| Apiaceae | *Bunium bulbocastanum* | Indicator | NA | NA | 0 | 1 | 0.00 |
| Apiaceae | *Bupleurum baldense* | Indicator | NA | NA | 0 | 1 | 0.00 |
| Apiaceae | *Bupleurum falcatum* | Indicator | NA | NA | 1 | 1 | 5.88 |
| Apiaceae | *Bupleurum ranunculoides* | Indicator | MPD | NA | 0 | 1 | 0.00 |
| Apiaceae | *Carum carvi* | Indicator | MPD | NA | 1 | 1 | 29.41 |
| Apiaceae | *Carum heldreichii* | Indicator | NA | NA | 0 | 0 | 0.00 |
| Apiaceae | *Carum verticillatum* | Indicator | NA | NA | 0 | 1 | 0.00 |
| Apiaceae | *Chaerophyllum aureum* | Indicator | MPD | NA | 1 | 0 | 11.76 |
| Apiaceae | *Chaerophyllum hirsutum* | Indicator | PD | NA | 0 | 1 | 0.00 |
| Apiaceae | *Chaerophyllum villarsii* | Indicator | NA | NA | 0 | 0 | 0.00 |
| Apiaceae | *Conopodium majus* | Indicator | MPD | NA | 1 | 1 | 11.76 |
| Apiaceae | *Conopodium pyrenaeum* | Indicator | NA | NA | 0 | 0 | 0.00 |
| Apiaceae | *Daucus carota* | Indicator | MD | NA | 1 | 1 | 58.82 |
| Apiaceae | *Dethawia splendens* | Indicator | NA | NA | 0 | 0 | 0.00 |
| Apiaceae | *Endressia pyrenaica* | Indicator | NA | NA | 0 | 0 | 0.00 |
| Apiaceae | *Eryngium alpinum* | Protected | NA | 92/43/CEE | 0 | 0 | 0.00 |
| Apiaceae | *Eryngium amethystinum* | Indicator | NA | NA | 0 | 0 | 0.00 |
| Apiaceae | *Eryngium bourgatii* | Indicator | NA | NA | 0 | 0 | 0.00 |
| Apiaceae | *Eryngium campestre* | Indicator | NA | NA | 1 | 0 | 23.53 |
| Apiaceae | *Falcaria vulgaris* | Indicator | MPD | NA | 1 | 1 | 11.76 |
| Apiaceae | *Heracleum austriacum* | Indicator | NA | NA | 0 | 0 | 0.00 |
| Apiaceae | *Heracleum sphondylium* | Indicator | MPD | NA | 1 | 1 | 29.41 |
| Apiaceae | *Laserpitium latifolium* | Indicator | NA | NA | 1 | 0 | 5.88 |
| Apiaceae | *Laserpitium nestleri* | Indicator | NA | NA | 0 | 0 | 0.00 |
| Apiaceae | *Laserpitium siler* | Indicator | NA | NA | 0 | 0 | 0.00 |
| Apiaceae | *Ligusticum mutellina* | Indicator | NA | NA | 1 | 1 | 5.88 |
| Apiaceae | *Ligusticum mutellinoides* | Indicator | NA | NA | 0 | 0 | 0.00 |
| Apiaceae | *Meum athamanticum* | Indicator | NA | NA | 1 | 1 | 11.76 |
| Apiaceae | *Oenanthe pimpinelloides* | Indicator | NA | NA | 0 | 0 | 0.00 |
| Apiaceae | *Orlaya grandiflora* | Indicator | NA | NA | 1 | 0 | 11.76 |
| Apiaceae | *Pastinaca sativa* | Indicator | MPD | NA | 1 | 1 | 29.41 |
| Apiaceae | *Peucedanum cervaria* | Indicator | NA | NA | 1 | 1 | 5.88 |
| Apiaceae | *Peucedanum oreoselinum* | Indicator | NA | NA | 1 | 1 | 17.65 |
| Apiaceae | *Pimpinella major* | Indicator | PD | NA | 1 | 1 | 29.41 |
| Apiaceae | *Pimpinella saxifraga* | Indicator | NA | NA | 1 | 1 | 41.18 |
| Apiaceae | *Pimpinella siifolia* | Indicator | NA | NA | 0 | 0 | 0.00 |
| Apiaceae | *Rouya polygama* | Protected | NA | 92/43/CEE | 0 | 0 | 0.00 |
| Apiaceae | *Selinum pyrenaeum* | Indicator | NA | NA | 0 | 0 | 0.00 |
| Apiaceae | *Seseli annuum* | Indicator | NA | NA | 1 | 0 | 5.88 |
| Apiaceae | *Seseli cantabricum* | Indicator | NA | NA | 0 | 0 | 0.00 |
| Apiaceae | *Seseli libanotis* | Indicator | NA | NA | 0 | 1 | 0.00 |
| Apiaceae | *Seseli montanum* | Indicator | NA | NA | 0 | 0 | 0.00 |
| Apiaceae | *Seseli nanum* | Indicator | NA | NA | 0 | 0 | 0.00 |
| Apiaceae | *Thorella verticillatoinundata* | Protected | NA | 92/43/CEE | 0 | 0 | 0.00 |
| Apiaceae | *Trinia dalechampii* | Indicator | NA | NA | 0 | 0 | 0.00 |
| Apiaceae | *Trinia glauca* | Indicator | MPD | NA | 0 | 1 | 0.00 |
| Apocynaceae | *Vincetoxicum hirundinaria* | Indicator | NA | NA | 1 | 1 | 11.76 |
| Asparagaceae | *Anthericum liliago* | Indicator | NA | NA | 1 | 0 | 17.65 |
| Asparagaceae | *Anthericum ramosum* | Indicator | NA | NA | 1 | 1 | 17.65 |
| Asparagaceae | *Aphyllanthes monspeliensis* | Indicator | NA | NA | 0 | 0 | 0.00 |
| Asparagaceae | *Hyacinthoides mauritanica* | Protected | NA | 92/43/CEE | 0 | 0 | 0.00 |
| Asparagaceae | *Muscari botryoides* | Indicator | NA | NA | 0 | 0 | 0.00 |
| Asparagaceae | *Muscari neglectum* | Indicator | MPD | NA | 0 | 1 | 0.00 |
| Asparagaceae | *Muscari racemosum* | Indicator | NA | NA | 0 | 0 | 0.00 |
| Asparagaceae | *Ornithogalum reverchonii* | Protected | NA | 92/43/CEE | 0 | 0 | 0.00 |
| Asparagaceae | *Ornithogalum umbellatum* | Indicator | NA | NA | 0 | 0 | 0.00 |
| Asparagaceae | *Paradisea liliastrum* | Indicator | NA | NA | 0 | 0 | 0.00 |
| Asparagaceae | *Polygonatum odoratum* | Indicator | MPD | NA | 1 | 1 | 5.88 |
| Asparagaceae | *Polygonatum verticillatum* | Indicator | MPD | NA | 1 | 1 | 5.88 |
| Asparagaceae | *Scilla ramburei* | Protected | NA | 92/43/CEE | 0 | 0 | 0.00 |
| Asparagaceae | *Scilla verna* | Indicator | NA | NA | 0 | 0 | 0.00 |
| Boraginaceae | *Cynoglossum magellense* | Indicator | NA | NA | 0 | 0 | 0.00 |
| Boraginaceae | *Echium vulgare* | Indicator | PD | NA | 1 | 1 | 64.71 |
| Boraginaceae | *Myosotis alpestris* | Indicator | PD | NA | 1 | 1 | 11.76 |
| Boraginaceae | *Myosotis ambigens* | Indicator | NA | NA | 0 | 0 | 0.00 |
| Boraginaceae | *Myosotis arvensis* | Indicator | NA | NA | 1 | 1 | 29.41 |
| Boraginaceae | *Myosotis discolor* | Indicator | NA | NA | 0 | 1 | 0.00 |
| Boraginaceae | *Myosotis ramosissima* | Indicator | PD | NA | 0 | 1 | 0.00 |
| Boraginaceae | *Myosotis scorpioides* | Indicator | NA | NA | 1 | 1 | 23.53 |
| Boraginaceae | *Myosotis sylvatica* | Indicator | NA | NA | 1 | 1 | 35.29 |
| Boraginaceae | *Omphalodes littoralis* | Protected | NA | 92/43/CEE | 0 | 0 | 0.00 |
| Boraginaceae | *Onosma bubanii* | Indicator | NA | NA | 0 | 0 | 0.00 |
| Brassicaceae | *Alyssum montanum* | Indicator | NA | NA | 1 | 1 | 5.88 |
| Brassicaceae | *Arabidopsis arenosa* | Indicator | NA | NA | 0 | 0 | 0.00 |
| Brassicaceae | *Arabidopsis halleri* | Indicator | NA | NA | 0 | 0 | 0.00 |
| Brassicaceae | *Arabidopsis thaliana* | Indicator | ND | NA | 1 | 1 | 5.88 |
| Brassicaceae | *Arabis alpina* | Indicator | NA | NA | 0 | 1 | 0.00 |
| Brassicaceae | *Arabis ciliata* | Indicator | NA | NA | 0 | 0 | 0.00 |
| Brassicaceae | *Arabis collina* | Indicator | NA | NA | 0 | 0 | 0.00 |
| Brassicaceae | *Arabis hirsuta* | Indicator | NA | NA | 1 | 1 | 11.76 |
| Brassicaceae | *Aurinia saxatilis* | Indicator | PD | NA | 1 | 1 | 5.88 |
| Brassicaceae | *Biscutella laevigata* | Indicator | PD | NA | 1 | 1 | 5.88 |
| Brassicaceae | *Biscutella valentina* | Indicator | NA | NA | 1 | 0 | 5.88 |
| Brassicaceae | *Capsella bursa pastoris* | Indicator | PD | NA | 1 | 1 | 52.94 |
| Brassicaceae | *Cardamine hirsuta* | Indicator | PD | NA | 0 | 1 | 0.00 |
| Brassicaceae | *Cardamine pratensis* | Indicator | NA | NA | 1 | 1 | 29.41 |
| Brassicaceae | *Cardamine resedifolia* | Indicator | NA | NA | 0 | 0 | 0.00 |
| Brassicaceae | *Cochlearia officinalis* | Indicator | ND | NA | 0 | 1 | 0.00 |
| Brassicaceae | *Draba aizoides* | Indicator | PD | NA | 0 | 1 | 0.00 |
| Brassicaceae | *Draba carinthiaca* | Indicator | PD | NA | 0 | 0 | 0.00 |
| Brassicaceae | *Draba fladnizensis* | Indicator | PD | NA | 0 | 0 | 0.00 |
| Brassicaceae | *Draba incana* | Indicator | PD | NA | 0 | 1 | 0.00 |
| Brassicaceae | *Draba norvegica* | Indicator | NA | NA | 0 | 1 | 0.00 |
| Brassicaceae | *Draba siliquosa* | Indicator | PD | NA | 0 | 0 | 0.00 |
| Brassicaceae | *Erysimum duriaei* | Indicator | NA | NA | 0 | 0 | 0.00 |
| Brassicaceae | *Erysimum pseudorhaeticum* | Indicator | NA | NA | 0 | 0 | 0.00 |
| Brassicaceae | *Iberis sempervirens* | Indicator | NA | NA | 1 | 0 | 5.88 |
| Brassicaceae | *Murbeckiella pinnatifida* | Indicator | NA | NA | 0 | 0 | 0.00 |
| Brassicaceae | *Pritzelago alpina* | Indicator | PD | NA | 0 | 1 | 0.00 |
| Brassicaceae | *Thlaspi alpestre* | Indicator | NA | NA | 0 | 1 | 0.00 |
| Brassicaceae | *Thlaspi caerulescens* | Indicator | NA | NA | 0 | 1 | 0.00 |
| Brassicaceae | *Thlaspi praecox* | Indicator | NA | NA | 0 | 0 | 0.00 |
| Campanulaceae | *Campanula alpina* | Indicator | NA | NA | 0 | 0 | 0.00 |
| Campanulaceae | *Campanula barbata* | Indicator | MPD | NA | 1 | 1 | 5.88 |
| Campanulaceae | *Campanula bohemica* | Indicator | MPD | NA | 1 | 0 | 5.88 |
| Campanulaceae | *Campanula carnica* | Indicator | NA | NA | 0 | 0 | 0.00 |
| Campanulaceae | *Campanula cochleariifolia* | Indicator | NA | NA | 0 | 0 | 0.00 |
| Campanulaceae | *Campanula ficarioides* | Indicator | NA | NA | 0 | 0 | 0.00 |
| Campanulaceae | *Campanula glomerata* | Indicator | MPD | NA | 1 | 1 | 35.29 |
| Campanulaceae | *Campanula herminii* | Indicator | NA | NA | 0 | 0 | 0.00 |
| Campanulaceae | *Campanula hispanica* | Indicator | NA | NA | 0 | 0 | 0.00 |
| Campanulaceae | *Campanula micrantha* | Indicator | NA | NA | 0 | 0 | 0.00 |
| Campanulaceae | *Campanula patula* | Indicator | NA | NA | 1 | 1 | 17.65 |
| Campanulaceae | *Campanula rapunculoides* | Indicator | MPD | NA | 1 | 1 | 41.18 |
| Campanulaceae | *Campanula rotundifolia* | Indicator | MD | NA | 1 | 1 | 52.94 |
| Campanulaceae | *Campanula scheuchzeri* | Indicator | MPD | NA | 1 | 1 | 5.88 |
| Campanulaceae | *Campanula serrata* | Indicator | NA | NA | 0 | 0 | 0.00 |
| Campanulaceae | *Edraianthus graminifolius* | Indicator | NA | NA | 0 | 0 | 0.00 |
| Campanulaceae | *Jasione cavanillesii* | Indicator | NA | NA | 0 | 0 | 0.00 |
| Campanulaceae | *Jasione crispa* | Indicator | NA | NA | 0 | 0 | 0.00 |
| Campanulaceae | *Jasione laevis* | Indicator | NA | NA | 0 | 0 | 0.00 |
| Campanulaceae | *Jasione montana* | Indicator | NA | NA | 1 | 0 | 35.29 |
| Campanulaceae | *Jasione sessiliflora* | Protected | NA | 92/43/CEE | 0 | 0 | 0.00 |
| Campanulaceae | *Phyteuma betonicifolium* | Indicator | NA | NA | 1 | 0 | 5.88 |
| Campanulaceae | *Phyteuma confusum* | Indicator | NA | NA | 0 | 0 | 0.00 |
| Campanulaceae | *Phyteuma globulariifolium* | Indicator | NA | NA | 0 | 0 | 0.00 |
| Campanulaceae | *Phyteuma hemisphaericum* | Indicator | MPD | NA | 0 | 0 | 0.00 |
| Campanulaceae | *Phyteuma nigrum* | Indicator | PD | NA | 1 | 1 | 11.76 |
| Campanulaceae | *Phyteuma orbiculare* | Indicator | MPD | NA | 1 | 1 | 11.76 |
| Campanulaceae | *Phyteuma ovatum* | Indicator | NA | NA | 0 | 0 | 0.00 |
| Campanulaceae | *Phyteuma persicifolium* | Indicator | NA | NA | 0 | 0 | 0.00 |
| Campanulaceae | *Phyteuma scheutzeri* | Indicator | NA | NA | 0 | 0 | 0.00 |
| Campanulaceae | *Phyteuma spicatum* | Indicator | MPD | NA | 1 | 1 | 17.65 |
| Caprifoliaceae | *Knautia arvensis* | Indicator | PD | NA | 1 | 1 | 58.82 |
| Caprifoliaceae | *Knautia dipsacifolia* | Indicator | NA | NA | 1 | 1 | 11.76 |
| Caprifoliaceae | *Knautia drymeia* | Indicator | NA | NA | 0 | 0 | 0.00 |
| Caprifoliaceae | *Knautia longifolia* | Indicator | NA | NA | 0 | 0 | 0.00 |
| Caprifoliaceae | *Knautia nevadensis* | Indicator | NA | NA | 0 | 0 | 0.00 |
| Caprifoliaceae | *Knautia purpurea* | Indicator | NA | NA | 0 | 0 | 0.00 |
| Caprifoliaceae | *Scabiosa columbaria* | Indicator | NA | NA | 1 | 1 | 52.94 |
| Caprifoliaceae | *Scabiosa lucida* | Indicator | PD | NA | 0 | 1 | 0.00 |
| Caprifoliaceae | *Scabiosa ochroleuca* | Indicator | PD | NA | 1 | 1 | 17.65 |
| Caprifoliaceae | *Succisa pratensis* | Indicator | NA | NA | 1 | 1 | 47.06 |
| Caprifoliaceae | *Valeriana celtica* | Indicator | NA | NA | 0 | 0 | 0.00 |
| Caprifoliaceae | *Valeriana montana* | Indicator | NA | NA | 0 | 0 | 0.00 |
| Caprifoliaceae | *Valeriana repens* | Indicator | NA | NA | 1 | 0 | 5.88 |
| Caprifoliaceae | *Valeriana saxatilis* | Indicator | NA | NA | 0 | 0 | 0.00 |
| Caprifoliaceae | *Valerianella locusta* | Indicator | NA | NA | 1 | 1 | 5.88 |
| Caryophyllaceae | *Agrostemma githago* | Indicator | NA | NA | 1 | 1 | 52.94 |
| Caryophyllaceae | *Arenaria ciliata* | Indicator | PD | NA | 0 | 1 | 0.00 |
| Caryophyllaceae | *Arenaria grandiflora* | Indicator | ND | NA | 1 | 0 | 5.88 |
| Caryophyllaceae | *Arenaria purpurascens* | Indicator | ND | NA | 0 | 0 | 0.00 |
| Caryophyllaceae | *Arenaria serpyllifolia* | Indicator | PD | NA | 1 | 1 | 5.88 |
| Caryophyllaceae | *Cerastium alpinum* | Indicator | ND | NA | 1 | 1 | 5.88 |
| Caryophyllaceae | *Cerastium arvense* | Indicator | NA | NA | 1 | 1 | 11.76 |
| Caryophyllaceae | *Cerastium fontanum* | Indicator | NA | NA | 1 | 1 | 5.88 |
| Caryophyllaceae | *Cerastium glomeratum* | Indicator | NA | NA | 1 | 1 | 5.88 |
| Caryophyllaceae | *Cerastium pedunculatum* | Indicator | PD | NA | 0 | 0 | 0.00 |
| Caryophyllaceae | *Cerastium tomentosum* | Indicator | NA | NA | 0 | 0 | 0.00 |
| Caryophyllaceae | *Dianthus alpinus* | Indicator | NA | NA | 0 | 0 | 0.00 |
| Caryophyllaceae | *Dianthus armeria* | Indicator | PD | NA | 1 | 1 | 29.41 |
| Caryophyllaceae | *Dianthus barbatus* | Indicator | NA | NA | 1 | 1 | 11.76 |
| Caryophyllaceae | *Dianthus borbasii* | Indicator | NA | NA | 0 | 0 | 0.00 |
| Caryophyllaceae | *Dianthus carthusianorum* | Indicator | NA | NA | 1 | 1 | 35.29 |
| Caryophyllaceae | *Dianthus deltoides* | Indicator | NA | NA | 1 | 1 | 47.06 |
| Caryophyllaceae | *Dianthus furcatus* | Indicator | NA | NA | 0 | 0 | 0.00 |
| Caryophyllaceae | *Dianthus glacialis* | Indicator | ND | NA | 0 | 0 | 0.00 |
| Caryophyllaceae | *Dianthus hyssopifolius* | Indicator | NA | NA | 1 | 1 | 5.88 |
| Caryophyllaceae | *Dianthus marizii* | Protected | NA | 92/43/CEE | 0 | 0 | 0.00 |
| Caryophyllaceae | *Dianthus pavonius* | Indicator | NA | NA | 0 | 0 | 0.00 |
| Caryophyllaceae | *Dianthus rupicola* | Protected | NA | 92/43/CEE | 0 | 0 | 0.00 |
| Caryophyllaceae | *Dianthus superbus* | Indicator | PD | NA | 1 | 1 | 11.76 |
| Caryophyllaceae | *Dianthus sylvestris* | Indicator | NA | NA | 1 | 1 | 5.88 |
| Caryophyllaceae | *Gypsophila repens* | Indicator | PD | NA | 0 | 1 | 0.00 |
| Caryophyllaceae | *Herniaria algarvica* | Protected | NA | 92/43/CEE | 0 | 0 | 0.00 |
| Caryophyllaceae | *Herniaria berlengiana* | Protected | NA | 92/43/CEE | 0 | 0 | 0.00 |
| Caryophyllaceae | *Herniaria maritima* | Protected | NA | 92/43/CEE | 0 | 0 | 0.00 |
| Caryophyllaceae | *Minuartia recurva* | Indicator | NA | NA | 0 | 1 | 0.00 |
| Caryophyllaceae | *Minuartia sedoides* | Indicator | PD | NA | 0 | 1 | 0.00 |
| Caryophyllaceae | *Minuartia verna* | Indicator | PD | NA | 0 | 1 | 0.00 |
| Caryophyllaceae | *Paronychia kapela* | Indicator | NA | NA | 0 | 0 | 0.00 |
| Caryophyllaceae | *Paronychia polygonifolia* | Indicator | NA | NA | 0 | 0 | 0.00 |
| Caryophyllaceae | *Petrorhagia saxifraga* | Indicator | ND | NA | 1 | 1 | 29.41 |
| Caryophyllaceae | *Sagina pilifera* | Indicator | NA | NA | 0 | 0 | 0.00 |
| Caryophyllaceae | *Sagina saginoides* | Indicator | NA | NA | 0 | 1 | 0.00 |
| Caryophyllaceae | *Saponaria caespitosa* | Indicator | NA | NA | 0 | 0 | 0.00 |
| Caryophyllaceae | *Saponaria ocymoides* | Indicator | NA | NA | 1 | 0 | 5.88 |
| Caryophyllaceae | *Saponaria pumila* | Indicator | NA | NA | 0 | 0 | 0.00 |
| Caryophyllaceae | *Saponaria pumilio* | Indicator | NA | NA | 0 | 1 | 0.00 |
| Caryophyllaceae | *Scleranthus perennis* | Indicator | NA | NA | 0 | 0 | 0.00 |
| Caryophyllaceae | *Silene acaulis* | Indicator | PD | NA | 0 | 1 | 0.00 |
| Caryophyllaceae | *Silene alpestris* | Indicator | NA | NA | 0 | 1 | 0.00 |
| Caryophyllaceae | *Silene ciliata* | Indicator | PD | NA | 0 | 1 | 0.00 |
| Caryophyllaceae | *Silene dioica* | Indicator | PD | NA | 1 | 1 | 58.82 |
| Caryophyllaceae | *Silene flos-cuculi* | Indicator | PD | NA | 1 | 1 | 64.71 |
| Caryophyllaceae | *Silene latifolia* | Indicator | NA | NA | 1 | 1 | 29.41 |
| Caryophyllaceae | *Silene multicaulis* | Indicator | NA | NA | 0 | 1 | 0.00 |
| Caryophyllaceae | *Silene nutans* | Indicator | NA | NA | 1 | 1 | 29.41 |
| Caryophyllaceae | *Silene otites* | Indicator | NA | NA | 1 | 1 | 5.88 |
| Caryophyllaceae | *Silene roemeri* | Indicator | NA | NA | 0 | 0 | 0.00 |
| Caryophyllaceae | *Silene rupestris* | Indicator | NA | NA | 0 | 1 | 0.00 |
| Caryophyllaceae | *Silene scabriflora* | Protected | NA | 92/43/CEE | 0 | 0 | 0.00 |
| Caryophyllaceae | *Silene uniflora* | Indicator | NA | NA | 1 | 1 | 11.76 |
| Caryophyllaceae | *Silene vallesia* | Indicator | NA | NA | 0 | 1 | 0.00 |
| Caryophyllaceae | *Silene vulgaris* | Indicator | PD | NA | 1 | 1 | 76.47 |
| Caryophyllaceae | *Stellaria graminea* | Indicator | PD | NA | 1 | 1 | 23.53 |
| Caryophyllaceae | *Stellaria media* | Indicator | NA | NA | 1 | 1 | 5.88 |
| Cistaceae | *Halimium verticillatum* | Protected | NA | 92/43/CEE | 0 | 0 | 0.00 |
| Cistaceae | *Helianthemum apenninum* | Indicator | NA | NA | 0 | 1 | 0.00 |
| Cistaceae | *Helianthemum canum* | Indicator | PY | NA | 0 | 0 | 0.00 |
| Cistaceae | *Helianthemum caput-felis* | Protected | NA | 92/43/CEE | 0 | 1 | 0.00 |
| Cistaceae | *Helianthemum nummularium* | Indicator | NA | NA | 1 | 1 | 11.76 |
| Cistaceae | *Helianthemum oelandicum* | Indicator | PY | NA | 0 | 0 | 0.00 |
| Colchicaceae | *Androcymbium europaeum* | Protected | NA | 92/43/CEE | 0 | 1 | 0.00 |
| Colchicaceae | *Coincya rupestris* | Protected | PD | 92/43/CEE | 0 | 1 | 0.00 |
| Colchicaceae | *Colchicum autumnale* | Indicator | NA | NA | 1 | 0 | 17.65 |
| Colchicaceae | *Colchicum corsicum* | Protected | NA | 92/43/CEE | 0 | 0 | 0.00 |
| Colchicaceae | *Colchicum montanum* | Indicator | NA | NA | 0 | 0 | 0.00 |
| Compositae | *Achillea clavennae* | Indicator | NA | NA | 0 | 0 | 0.00 |
| Compositae | *Achillea collina* | Indicator | NA | NA | 1 | 1 | 5.88 |
| Compositae | *Achillea erbarotta* | Indicator | PD | NA | 1 | 1 | 5.88 |
| Compositae | *Achillea millefolium* | Indicator | PD | NA | 1 | 1 | 88.24 |
| Compositae | *Achillea pannonica* | Indicator | NA | NA | 1 | 0 | 5.88 |
| Compositae | *Achillea setacea* | Indicator | NA | NA | 0 | 0 | 0.00 |
| Compositae | *Achillea stricta* | Indicator | NA | NA | 0 | 0 | 0.00 |
| Compositae | *Achillea virescens* | Indicator | NA | NA | 0 | 0 | 0.00 |
| Compositae | *Adenostyles alpina* | Indicator | NA | NA | 0 | 0 | 0.00 |
| Compositae | *Antennaria carpatica* | Indicator | ND | NA | 0 | 0 | 0.00 |
| Compositae | *Antennaria dioica* | Indicator | ND | NA | 1 | 1 | 5.88 |
| Compositae | *Arnica montana* | Indicator | PD | NA | 1 | 1 | 23.53 |
| Compositae | *Artemisia absinthium* | Indicator | PD | NA | 1 | 1 | 29.41 |
| Compositae | *Artemisia alba* | Indicator | NA | NA | 0 | 1 | 0.00 |
| Compositae | *Artemisia atrata* | Indicator | NA | NA | 0 | 0 | 0.00 |
| Compositae | *Artemisia austriaca* | Indicator | NA | NA | 0 | 0 | 0.00 |
| Compositae | *Artemisia campestris* | Indicator | PD | NA | 1 | 1 | 5.88 |
| Compositae | *Artemisia umbelliformis* | Indicator | ND | NA | 0 | 0 | 0.00 |
| Compositae | *Artemisia vulgaris* | Indicator | PD | NA | 1 | 1 | 11.76 |
| Compositae | *Aster alpinus* | Indicator | ND | NA | 0 | 1 | 0.00 |
| Compositae | *Aster amellus* | Indicator | PD | NA | 1 | 1 | 23.53 |
| Compositae | *Aster pyrenaeus* | Protected | NA | 92/43/CEE | 0 | 0 | 0.00 |
| Compositae | *Bellidastrum michelii* | Indicator | NA | NA | 0 | 0 | 0.00 |
| Compositae | *Bellis perennis* | Indicator | PD | NA | 1 | 1 | 58.82 |
| Compositae | *Buphthalmum salicifolium* | Indicator | NA | NA | 1 | 0 | 29.41 |
| Compositae | *Carduncellus monspeliensis* | Indicator | NA | NA | 0 | 0 | 0.00 |
| Compositae | *Carduus carlinoides* | Indicator | NA | NA | 0 | 0 | 0.00 |
| Compositae | *Carduus defloratus* | Indicator | ND | NA | 1 | 1 | 5.88 |
| Compositae | *Carlina acanthifolia* | Indicator | NA | NA | 0 | 1 | 0.00 |
| Compositae | *Carlina acaulis* | Indicator | PD | NA | 1 | 1 | 11.76 |
| Compositae | *Carlina vulgaris* | Indicator | PD | NA | 1 | 1 | 29.41 |
| Compositae | *Catananche caerulea* | Indicator | NA | NA | 1 | 1 | 5.88 |
| Compositae | *Centaurea ambigua* | Indicator | NA | NA | 0 | 0 | 0.00 |
| Compositae | *Centaurea corymbosa* | Protected | NA | 92/43/CEE | 0 | 0 | 0.00 |
| Compositae | *Centaurea herminii* | Protected | NA | 92/43/CEE | 0 | 0 | 0.00 |
| Compositae | *Centaurea jacea* | Indicator | NA | NA | 1 | 1 | 41.18 |
| Compositae | *Centaurea nemoralis* | Indicator | NA | NA | 0 | 0 | 0.00 |
| Compositae | *Centaurea nervosa* | Indicator | NA | NA | 1 | 0 | 5.88 |
| Compositae | *Centaurea nigra* | Indicator | NA | NA | 1 | 1 | 17.65 |
| Compositae | *Centaurea nigrescens* | Indicator | NA | NA | 1 | 0 | 5.88 |
| Compositae | *Centaurea phrygia* | Indicator | NA | NA | 0 | 1 | 0.00 |
| Compositae | *Centaurea princeps* | Protected | NA | 92/43/CEE | 0 | 0 | 0.00 |
| Compositae | *Centaurea rothmalerana* | Protected | NA | 92/43/CEE | 0 | 0 | 0.00 |
| Compositae | *Centaurea scabiosa* | Indicator | NA | NA | 1 | 1 | 52.94 |
| Compositae | *Centaurea triumfetti* | Indicator | NA | NA | 1 | 1 | 5.88 |
| Compositae | *Cichorium intybus* | Indicator | NA | NA | 1 | 1 | 64.71 |
| Compositae | *Cirsium acaule* | Indicator | NA | NA | 1 | 1 | 5.88 |
| Compositae | *Cirsium arvense* | Indicator | PD | NA | 1 | 1 | 5.88 |
| Compositae | *Cirsium eriophorum* | Indicator | NA | NA | 1 | 0 | 23.53 |
| Compositae | *Cirsium erisithales* | Indicator | NA | NA | 0 | 0 | 0.00 |
| Compositae | *Cirsium oleraceum* | Indicator | NA | NA | 1 | 0 | 17.65 |
| Compositae | *Cirsium pannonicum* | Indicator | NA | NA | 1 | 0 | 5.88 |
| Compositae | *Crepis aurea* | Indicator | NA | NA | 1 | 0 | 5.88 |
| Compositae | *Crepis biennis* | Indicator | NA | NA | 1 | 1 | 23.53 |
| Compositae | *Crepis capillaris* | Indicator | NA | NA | 1 | 1 | 23.53 |
| Compositae | *Crepis conyzifolia* | Indicator | NA | NA | 1 | 1 | 5.88 |
| Compositae | *Crepis jacquinii* | Indicator | NA | NA | 0 | 0 | 0.00 |
| Compositae | *Crepis mollis* | Indicator | NA | NA | 0 | 1 | 0.00 |
| Compositae | *Crepis pyrenaica* | Indicator | NA | NA | 0 | 0 | 0.00 |
| Compositae | *Crepis terglouensis* | Indicator | NA | NA | 0 | 0 | 0.00 |
| Compositae | *Cyanus montanus* | Indicator | NA | NA | 1 | 0 | 5.88 |
| Compositae | *Cyanus segetum* | Indicator | NA | NA | 1 | 1 | 76.47 |
| Compositae | *Doronicum clusii* | Indicator | NA | NA | 0 | 0 | 0.00 |
| Compositae | *Doronicum columnae* | Indicator | NA | NA | 0 | 0 | 0.00 |
| Compositae | *Erigeron acer* | Indicator | ND | NA | 1 | 1 | 5.88 |
| Compositae | *Erigeron alpinus* | Indicator | NA | NA | 0 | 0 | 0.00 |
| Compositae | *Erigeron annuus* | Indicator | ND | NA | 1 | 1 | 5.88 |
| Compositae | *Erigeron epiroticus* | Indicator | NA | NA | 0 | 0 | 0.00 |
| Compositae | *Erigeron frigidus* | Protected | NA | 92/43/CEE | 0 | 0 | 0.00 |
| Compositae | *Erigeron uniflorus* | Indicator | ND | NA | 0 | 1 | 0.00 |
| Compositae | *Galatella linosyris* | Indicator | NA | NA | 1 | 1 | 11.76 |
| Compositae | *Galatella villosa* | Indicator | NA | NA | 0 | 0 | 0.00 |
| Compositae | *Glebionis segetum* | Indicator | NA | NA | 1 | 0 | 11.76 |
| Compositae | *Gnaphalium supinum* | Indicator | PD | NA | 0 | 1 | 0.00 |
| Compositae | *Gnaphalium sylvaticum* | Indicator | PD | NA | 1 | 1 | 5.88 |
| Compositae | *Helichrysum sibthorpii* | Protected | NA | 92/43/CEE | 0 | 0 | 0.00 |
| Compositae | *Hieracium alpinum* | Indicator | PD | NA | 1 | 1 | 5.88 |
| Compositae | *Hieracium amplexicaule* | Indicator | NA | NA | 0 | 0 | 0.00 |
| Compositae | *Hieracium bifidum* | Indicator | NA | NA | 0 | 0 | 0.00 |
| Compositae | *Hieracium caesium* | Indicator | NA | NA | 0 | 0 | 0.00 |
| Compositae | *Hieracium cymosum* | Indicator | NA | NA | 1 | 0 | 5.88 |
| Compositae | *Hieracium glaciale* | Indicator | NA | NA | 0 | 0 | 0.00 |
| Compositae | *Hieracium hoppeanum* | Indicator | NA | NA | 0 | 0 | 0.00 |
| Compositae | *Hieracium iseranum* | Indicator | NA | NA | 0 | 0 | 0.00 |
| Compositae | *Hieracium lachenalii* | Indicator | NA | NA | 1 | 0 | 11.76 |
| Compositae | *Hieracium murorum* | Indicator | NA | NA | 1 | 0 | 17.65 |
| Compositae | *Hieracium piliferum* | Indicator | NA | NA | 0 | 0 | 0.00 |
| Compositae | *Hieracium praealtum* | Indicator | NA | NA | 1 | 0 | 5.88 |
| Compositae | *Hieracium villosum* | Indicator | NA | NA | 0 | 0 | 0.00 |
| Compositae | *Homogyne alpina* | Indicator | PD | NA | 0 | 1 | 0.00 |
| Compositae | *Homogyne discolor* | Indicator | NA | NA | 0 | 0 | 0.00 |
| Compositae | *Hypochaeris radicata* | Indicator | ND | NA | 1 | 1 | 17.65 |
| Compositae | *Hypochaeris uniflora* | Indicator | NA | NA | 1 | 0 | 5.88 |
| Compositae | *Inula ensifolia* | Indicator | PY | NA | 1 | 1 | 5.88 |
| Compositae | *Jacobaea incana* | Indicator | PD | NA | 0 | 1 | 0.00 |
| Compositae | *Jacobaea uniflora* | Indicator | NA | NA | 0 | 0 | 0.00 |
| Compositae | *Jacobaea vulgaris* | Indicator | ND | NA | 1 | 1 | 23.53 |
| Compositae | *Jacobea adonidifolia* | Indicator | NA | NA | 0 | 0 | 0.00 |
| Compositae | *Jurinea cyanoides* | Protected | NA | 92/43/CEE | 1 | 0 | 5.88 |
| Compositae | *Jurinea humilis* | Indicator | NA | NA | 0 | 0 | 0.00 |
| Compositae | *Leontodon boryi* | Protected | NA | 92/43/CEE | 0 | 0 | 0.00 |
| Compositae | *Leontodon crispus* | Indicator | NA | NA | 0 | 0 | 0.00 |
| Compositae | *Leontodon hispidus* | Indicator | ND | NA | 1 | 1 | 23.53 |
| Compositae | *Leontodon siculus* | Protected | NA | 92/43/CEE | 0 | 0 | 0.00 |
| Compositae | *Leontopodium nivale* | Indicator | PD | NA | 1 | 1 | 5.88 |
| Compositae | *Leucanthemopsis alpina* | Indicator | NA | NA | 0 | 1 | 0.00 |
| Compositae | *Leucanthemum adustum* | Indicator | NA | NA | 1 | 0 | 5.88 |
| Compositae | *Leucanthemum atratum* | Indicator | NA | NA | 0 | 0 | 0.00 |
| Compositae | *Leucanthemum ircutianum* | Indicator | NA | NA | 1 | 0 | 5.88 |
| Compositae | *Leucanthemum maximum* | Indicator | NA | NA | 0 | 0 | 0.00 |
| Compositae | *Leucanthemum tridactylites* | Indicator | NA | NA | 0 | 0 | 0.00 |
| Compositae | *Leucanthemum vulgare* | Indicator | NA | NA | 1 | 1 | 52.94 |
| Compositae | *Ligularia sibirica* | Protected | PD | 92/43/CEE | 1 | 1 | 5.88 |
| Compositae | *Picris willkommii* | Protected | NA | 92/43/CEE | 0 | 0 | 0.00 |
| Compositae | *Pilosella aurantiaca* | Indicator | NA | NA | 1 | 1 | 23.53 |
| Compositae | *Pilosella breviscapa* | Indicator | NA | NA | 0 | 0 | 0.00 |
| Compositae | *Pilosella lactucella* | Indicator | NA | NA | 1 | 0 | 11.76 |
| Compositae | *Pilosella officinarum* | Indicator | NA | NA | 1 | 1 | 11.76 |
| Compositae | *Pilosella peleteriana* | Indicator | NA | NA | 0 | 0 | 0.00 |
| Compositae | *Pilosella piloselloides* | Indicator | NA | NA | 0 | 0 | 0.00 |
| Compositae | *Podospermum purpureum* | Indicator | NA | NA | 0 | 0 | 0.00 |
| Compositae | *Rhaponticum longifolium* | Protected | NA | 92/43/CEE | 0 | 0 | 0.00 |
| Compositae | *Santolina semidentata* | Protected | NA | 92/43/CEE | 0 | 0 | 0.00 |
| Compositae | *Saussurea alpina* | Indicator | PD | NA | 0 | 1 | 0.00 |
| Compositae | *Scorzonera aristata* | Indicator | NA | NA | 0 | 0 | 0.00 |
| Compositae | *Scorzonera humilis* | Indicator | NA | NA | 1 | 1 | 5.88 |
| Compositae | *Scorzoneroides autumnalis* | Indicator | NA | NA | 1 | 1 | 35.29 |
| Compositae | *Scorzoneroides cichoriacea* | Indicator | NA | NA | 0 | 1 | 0.00 |
| Compositae | *Scorzoneroides helvetica* | Indicator | PD | NA | 0 | 0 | 0.00 |
| Compositae | *Scorzoneroides microcephala* | Protected | NA | 92/43/CEE | 0 | 0 | 0.00 |
| Compositae | *Scorzoneroides pyrenaica* | Indicator | NA | NA | 0 | 0 | 0.00 |
| Compositae | *Senecio abrotanifolius* | Indicator | NA | NA | 0 | 0 | 0.00 |
| Compositae | *Senecio doronicum* | Indicator | NA | NA | 0 | 0 | 0.00 |
| Compositae | *Senecio nemorensis* | Indicator | NA | NA | 0 | 0 | 0.00 |
| Compositae | *Senecio nevadensis* | Protected | NA | 92/43/CEE | 0 | 0 | 0.00 |
| Compositae | *Senecio pyrenaicus* | Indicator | NA | NA | 0 | 0 | 0.00 |
| Compositae | *Senecio scopolii* | Indicator | NA | NA | 0 | 0 | 0.00 |
| Compositae | *Solidago virgaurea* | Indicator | ND | NA | 1 | 1 | 23.53 |
| Compositae | *Tanacetum corymbosum* | Indicator | NA | NA | 1 | 1 | 23.53 |
| Compositae | *Taraxacum apenninum* | Indicator | NA | NA | 0 | 0 | 0.00 |
| Compositae | *Taraxacum campylodes* | Indicator | ND | NA | 1 | 1 | 11.76 |
| Compositae | *Taraxacum pyrenaicum* | Indicator | NA | NA | 0 | 0 | 0.00 |
| Compositae | *Tephroseris elodes* | Protected | NA | 92/43/CEE | 0 | 0 | 0.00 |
| Compositae | *Tragopogon pratensis* | Indicator | NA | NA | 1 | 1 | 5.88 |
| Compositae | *Tripolium sorrentinoi* | Protected | NA | 92/43/CEE | 0 | 0 | 0.00 |
| Compositae | *Tussilago farfara* | Indicator | NA | NA | 1 | 1 | 11.76 |
| Convolvulaceae | *Convolvulus arvensis* | Indicator | PY | NA | 1 | 1 | 11.76 |
| Crassulaceae | *Sedum acre* | Indicator | NA | NA | 1 | 1 | 47.06 |
| Crassulaceae | *Sedum album* | Indicator | NA | NA | 1 | 1 | 41.18 |
| Crassulaceae | *Sedum alpestre* | Indicator | NA | NA | 0 | 0 | 0.00 |
| Crassulaceae | *Sedum anglicum* | Indicator | NA | NA | 1 | 1 | 5.88 |
| Crassulaceae | *Sedum atratum* | Indicator | NA | NA | 0 | 0 | 0.00 |
| Crassulaceae | *Sedum brevifolium* | Indicator | NA | NA | 0 | 0 | 0.00 |
| Crassulaceae | *Sedum roseum* | Indicator | PD | NA | 0 | 1 | 0.00 |
| Crassulaceae | *Sedum rupestre* | Indicator | NA | NA | 1 | 1 | 17.65 |
| Crassulaceae | *Sedum sexangulare* | Indicator | NA | NA | 1 | 0 | 23.53 |
| Crassulaceae | *Sempervivum arachnoideum* | Indicator | NA | NA | 0 | 0 | 0.00 |
| Crassulaceae | *Sempervivum montanum* | Indicator | ND | NA | 0 | 0 | 0.00 |
| Crassulaceae | *Sempervivum tectorum* | Indicator | NA | NA | 1 | 0 | 5.88 |
| Cruciferae | *Biscutella neustriaca* | Protected | NA | 92/43/CEE | 0 | 0 | 0.00 |
| Cruciferae | *Diplotaxis ibicensis* | Protected | NA | 92/43/CEE | 0 | 0 | 0.00 |
| Cruciferae | *Sisymbrium supinum* | Protected | NA | 92/43/CEE | 0 | 0 | 0.00 |
| Cupressaceae | *Juniperus communis* | Indicator | PD | NA | 1 | 1 | 5.88 |
| Cyperaceae | *Carex arenaria* | Indicator | CD | NA | 0 | 1 | 0.00 |
| Cyperaceae | *Carex atrata* | Indicator | PD | NA | 0 | 1 | 0.00 |
| Cyperaceae | *Carex bigelowii* | Indicator | PD | NA | 0 | 1 | 0.00 |
| Cyperaceae | *Carex binervis* | Indicator | NA | NA | 0 | 0 | 0.00 |
| Cyperaceae | *Carex brevicollis* | Indicator | ND | NA | 0 | 0 | 0.00 |
| Cyperaceae | *Carex brunnescens* | Indicator | NA | NA | 0 | 0 | 0.00 |
| Cyperaceae | *Carex capillaris* | Indicator | PD | NA | 0 | 1 | 0.00 |
| Cyperaceae | *Carex caryophyllea* | Indicator | PD | NA | 1 | 1 | 5.88 |
| Cyperaceae | *Carex curvula* | Indicator | PD | NA | 0 | 1 | 0.00 |
| Cyperaceae | *Carex demissa* | Indicator | PD | NA | 1 | 1 | 5.88 |
| Cyperaceae | *Carex echinata* | Indicator | PD | NA | 1 | 1 | 5.88 |
| Cyperaceae | *Carex ericetorum* | Indicator | PD | NA | 1 | 1 | 5.88 |
| Cyperaceae | *Carex ferruginea* | Indicator | PD | NA | 0 | 1 | 0.00 |
| Cyperaceae | *Carex fimbriata* | Indicator | NA | NA | 0 | 0 | 0.00 |
| Cyperaceae | *Carex firma* | Indicator | PD | NA | 0 | 1 | 0.00 |
| Cyperaceae | *Carex flacca* | Indicator | PD | NA | 1 | 1 | 23.53 |
| Cyperaceae | *Carex flava* | Indicator | NA | NA | 1 | 1 | 11.76 |
| Cyperaceae | *Carex foetida* | Indicator | ND | NA | 0 | 0 | 0.00 |
| Cyperaceae | *Carex fuliginosa* | Indicator | NA | NA | 0 | 0 | 0.00 |
| Cyperaceae | *Carex hirta* | Indicator | PD | NA | 1 | 1 | 5.88 |
| Cyperaceae | *Carex humilis* | Indicator | NA | NA | 1 | 1 | 5.88 |
| Cyperaceae | *Carex kitaibeliana* | Indicator | NA | NA | 0 | 0 | 0.00 |
| Cyperaceae | *Carex leporina* | Indicator | PD | NA | 0 | 1 | 0.00 |
| Cyperaceae | *Carex macrolepis* | Indicator | NA | NA | 0 | 0 | 0.00 |
| Cyperaceae | *Carex montana* | Indicator | NA | NA | 1 | 0 | 5.88 |
| Cyperaceae | *Carex nigra* | Indicator | PD | NA | 0 | 1 | 0.00 |
| Cyperaceae | *Carex ornithopoda* | Indicator | ND | NA | 0 | 1 | 0.00 |
| Cyperaceae | *Carex ovalis* | Indicator | PD | NA | 0 | 1 | 0.00 |
| Cyperaceae | *Carex pairaei* | Indicator | NA | NA | 0 | 0 | 0.00 |
| Cyperaceae | *Carex pallescens* | Indicator | PD | NA | 1 | 1 | 5.88 |
| Cyperaceae | *Carex panicea* | Indicator | PD | NA | 1 | 1 | 5.88 |
| Cyperaceae | *Carex parviflora* | Indicator | ND | NA | 0 | 0 | 0.00 |
| Cyperaceae | *Carex pilulifera* | Indicator | PD | NA | 1 | 1 | 5.88 |
| Cyperaceae | *Carex pulicaris* | Indicator | NA | NA | 0 | 1 | 0.00 |
| Cyperaceae | *Carex pyrenaica* | Indicator | PD | NA | 0 | 1 | 0.00 |
| Cyperaceae | *Carex rupestris* | Indicator | NA | NA | 0 | 0 | 0.00 |
| Cyperaceae | *Carex sempervirens* | Indicator | PD | NA | 0 | 1 | 0.00 |
| Cyperaceae | *Carex trinervis* | Indicator | NA | NA | 0 | 0 | 0.00 |
| Cyperaceae | *Carex umbrosa* | Indicator | NA | NA | 0 | 0 | 0.00 |
| Cyperaceae | *Kobresia myosuroides* | Indicator | PD | NA | 0 | 1 | 0.00 |
| Ericaceae | *Erica herbacea* | Indicator | NA | NA | 0 | 0 | 0.00 |
| Ericaceae | *Erica vagans* | Indicator | PD | NA | 0 | 1 | 0.00 |
| Ericaceae | *Loiseleuria procumbens* | Indicator | PD | NA | 0 | 1 | 0.00 |
| Ericaceae | *Pyrola carpatica* | Indicator | NA | NA | 0 | 0 | 0.00 |
| Ericaceae | *Rhododendron ferrugineum* | Indicator | NA | NA | 0 | 1 | 0.00 |
| Ericaceae | *Rhododendron hirsutum* | Indicator | NA | NA | 0 | 0 | 0.00 |
| Ericaceae | *Rhodothamnus chamaecistus* | Indicator | NA | NA | 0 | 0 | 0.00 |
| Ericaceae | *Vaccinium myrtillus* | Indicator | PD | NA | 1 | 1 | 5.88 |
| Ericaceae | *Vaccinium uliginosum* | Indicator | PD | NA | 0 | 1 | 0.00 |
| Ericaceae | *Vaccinium vitis-idaea* | Indicator | PD | NA | 1 | 1 | 5.88 |
| Euphorbiaceae | *Euphorbia angulata* | Indicator | NA | NA | 0 | 0 | 0.00 |
| Euphorbiaceae | *Euphorbia cyparissias* | Indicator | NA | NA | 1 | 0 | 23.53 |
| Euphorbiaceae | *Euphorbia gasparrinii* | Indicator | NA | NA | 0 | 0 | 0.00 |
| Euphorbiaceae | *Euphorbia nevadensis* | Protected | NA | 92/43/CEE | 0 | 0 | 0.00 |
| Euphorbiaceae | *Mercurialis perennis* | Indicator | PD | NA | 1 | 1 | 17.65 |
| Gentianaceae | *Centaurium rigualii* | Protected | NA | 92/43/CEE | 0 | 0 | 0.00 |
| Gentianaceae | *Comastoma tenellum* | Indicator | MPD | NA | 0 | 1 | 0.00 |
| Gentianaceae | *Gentiana nivalis* | Indicator | MPD | NA | 0 | 1 | 0.00 |
| Gentianaceae | *Gentiana acaulis* | Indicator | MPD | NA | 0 | 1 | 0.00 |
| Gentianaceae | *Gentiana alpina* | Indicator | NA | NA | 0 | 0 | 0.00 |
| Gentianaceae | *Gentiana asclepiadea* | Indicator | MPD | NA | 0 | 1 | 0.00 |
| Gentianaceae | *Gentiana clusii* | Indicator | MPD | NA | 0 | 1 | 0.00 |
| Gentianaceae | *Gentiana dinarica* | Indicator | NA | NA | 0 | 0 | 0.00 |
| Gentianaceae | *Gentiana ligustica* | Protected | NA | 92/43/CEE | 0 | 1 | 0.00 |
| Gentianaceae | *Gentiana lutea* | Indicator | MPD | NA | 1 | 1 | 5.88 |
| Gentianaceae | *Gentiana nivalis* | Indicator | MPD | NA | 0 | 1 | 0.00 |
| Gentianaceae | *Gentiana occidentalis* | Indicator | NA | NA | 0 | 0 | 0.00 |
| Gentianaceae | *Gentiana pannonica* | Indicator | MPD | NA | 0 | 1 | 0.00 |
| Gentianaceae | *Gentiana pneumonanthe* | Indicator | MPD | NA | 1 | 1 | 5.88 |
| Gentianaceae | *Gentiana punctata* | Indicator | MPD | NA | 0 | 1 | 0.00 |
| Gentianaceae | *Gentiana purpurea* | Indicator | MPD | NA | 0 | 1 | 0.00 |
| Gentianaceae | *Gentiana verna* | Indicator | MPD | NA | 0 | 1 | 0.00 |
| Gentianaceae | *Gentianella anglica* | Protected | NA | 92/43/CEE | 0 | 1 | 0.00 |
| Gentianaceae | *Gentianella anisodonta* | Indicator | NA | NA | 0 | 0 | 0.00 |
| Gentianaceae | *Gentianella aspera* | Indicator | NA | NA | 0 | 0 | 0.00 |
| Gentianaceae | *Gentianella austriaca* | Indicator | NA | NA | 0 | 0 | 0.00 |
| Gentianaceae | *Gentianella campestris* | Indicator | MPD | NA | 0 | 1 | 0.00 |
| Gentianaceae | *Gentianella germanica* | Indicator | NA | NA | 0 | 1 | 0.00 |
| Gentianaceae | *Gentianella lutescens* | Indicator | NA | NA | 0 | 0 | 0.00 |
| Gentianaceae | *Swertia perennis* | Indicator | PD | NA | 0 | 1 | 0.00 |
| Geraniaceae | *Erodium astragaloides* | Protected | NA | 92/43/CEE | 0 | 0 | 0.00 |
| Geraniaceae | *Erodium cicutarium* | Indicator | PY | NA | 1 | 1 | 11.76 |
| Geraniaceae | *Geranium argenteum* | Indicator | PY | NA | 0 | 0 | 0.00 |
| Geraniaceae | *Geranium cinereum* | Indicator | NA | NA | 0 | 0 | 0.00 |
| Geraniaceae | *Geranium molle* | Indicator | PY | NA | 0 | 1 | 0.00 |
| Geraniaceae | *Geranium phaeum* | Indicator | NA | NA | 1 | 0 | 17.65 |
| Geraniaceae | *Geranium pratense* | Indicator | PY | NA | 1 | 1 | 52.94 |
| Geraniaceae | *Geranium sanguineum* | Indicator | PY | NA | 1 | 1 | 41.18 |
| Geraniaceae | *Geranium sylvaticum* | Indicator | PY | NA | 1 | 1 | 29.41 |
| Grossulariaceae | *Ribes sardoum* | Protected | NA | 92/43/CEE | 0 | 0 | 0.00 |
| Hypericaceae | *Hypericum aciferum* | Protected | NA | 92/43/CEE | 0 | 0 | 0.00 |
| Hypericaceae | *Hypericum linariifolium* | Indicator | NA | NA | 0 | 0 | 0.00 |
| Hypericaceae | *Hypericum maculatum* | Indicator | NA | NA | 1 | 1 | 23.53 |
| Hypericaceae | *Hypericum montanum* | Indicator | NA | NA | 1 | 1 | 23.53 |
| Hypericaceae | *Hypericum nummularium* | Indicator | NA | NA | 0 | 0 | 0.00 |
| Hypericaceae | *Hypericum perforatum* | Indicator | PD | NA | 1 | 1 | 58.82 |
| Hypericaceae | *Hypericum richeri* | Indicator | NA | NA | 0 | 0 | 0.00 |
| Hypericaceae | *Hypochaeris maculata* | Indicator | NA | NA | 1 | 1 | 11.76 |
| Iridaceae | *Crocus caeruleus* | Indicator | NA | NA | 0 | 0 | 0.00 |
| Iridaceae | *Crocus etruscus* | Protected | NA | 92/43/CEE | 0 | 0 | 0.00 |
| Iridaceae | *Crocus vernus* | Indicator | MPD | NA | 0 | 0 | 0.00 |
| Iridaceae | *Gladiolus illyricus* | Indicator | NA | NA | 0 | 1 | 0.00 |
| Iridaceae | *Iris latifolia* | Indicator | NA | NA | 0 | 0 | 0.00 |
| Iridaceae | *Iris marisca* | Protected | NA | 92/43/CEE | 0 | 0 | 0.00 |
| Juncaceae | *Juncus acutiflorus* | Indicator | NA | NA | 0 | 1 | 0.00 |
| Juncaceae | *Juncus jacquinii* | Indicator | NA | NA | 0 | 0 | 0.00 |
| Juncaceae | *Juncus squarrosus* | Indicator | NA | NA | 1 | 1 | 5.88 |
| Juncaceae | *Juncus trifidus* | Indicator | ND | NA | 0 | 1 | 0.00 |
| Juncaceae | *Juncus triglumis* | Indicator | NA | NA | 0 | 1 | 0.00 |
| Juncaceae | *Luzula alpinopilosa* | Indicator | NA | NA | 0 | 1 | 0.00 |
| Juncaceae | *Luzula campestris* | Indicator | PD | NA | 1 | 1 | 11.76 |
| Juncaceae | *Luzula lutea* | Indicator | NA | NA | 0 | 0 | 0.00 |
| Juncaceae | *Luzula luzuloides* | Indicator | NA | NA | 1 | 0 | 5.88 |
| Juncaceae | *Luzula multiflora* | Indicator | PD | NA | 1 | 1 | 11.76 |
| Juncaceae | *Luzula pediformis* | Indicator | NA | NA | 0 | 0 | 0.00 |
| Juncaceae | *Luzula pilosa* | Indicator | NA | NA | 0 | 1 | 0.00 |
| Juncaceae | *Luzula spicata* | Indicator | PD | NA | 0 | 1 | 0.00 |
| Juncaceae | *Luzula sudetica* | Indicator | NA | NA | 0 | 0 | 0.00 |
| Juncaceae | *Luzula sylvatica* | Indicator | ND | NA | 1 | 1 | 11.76 |
| Lamiaceae | *Ajuga pyramidalis* | Indicator | NA | NA | 0 | 0 | 0.00 |
| Lamiaceae | *Ajuga reptans* | Indicator | NA | NA | 1 | 1 | 52.94 |
| Lamiaceae | *Ajuga tenorei* | Indicator | NA | NA | 0 | 0 | 0.00 |
| Lamiaceae | *Clinopodium acinos* | Indicator | PY | NA | 1 | 1 | 11.76 |
| Lamiaceae | *Clinopodium alpinum* | Indicator | NA | NA | 0 | 0 | 0.00 |
| Lamiaceae | *Clinopodium vulgare* | Indicator | NA | NA | 1 | 1 | 23.53 |
| Lamiaceae | *Dracocephalum austriacum* | Protected | NA | 92/43/CEE | 0 | 0 | 0.00 |
| Lamiaceae | *Glechoma hederacea* | Indicator | PD | NA | 1 | 1 | 11.76 |
| Lamiaceae | *Horminum pyrenaicum* | Indicator | PD | NA | 0 | 1 | 0.00 |
| Lamiaceae | *Lamium album* | Indicator | NA | NA | 1 | 0 | 17.65 |
| Lamiaceae | *Lamium maculatum* | Indicator | NA | NA | 1 | 0 | 17.65 |
| Lamiaceae | *Lamium purpureum* | Indicator | PD | NA | 1 | 1 | 11.76 |
| Lamiaceae | *Lavandula angustifolia* | Indicator | PD | NA | 1 | 1 | 11.76 |
| Lamiaceae | *Nepeta sphaciotica* | Protected | NA | 92/43/CEE | 0 | 0 | 0.00 |
| Lamiaceae | *Origanum vulgare* | Indicator | NA | NA | 1 | 1 | 35.29 |
| Lamiaceae | *Prunella grandiflora* | Indicator | ND | NA | 1 | 1 | 23.53 |
| Lamiaceae | *Prunella laciniata* | Indicator | NA | NA | 1 | 1 | 17.65 |
| Lamiaceae | *Prunella vulgaris* | Indicator | PD | NA | 1 | 1 | 76.47 |
| Lamiaceae | *Salvia nemorosa* | Indicator | PD | NA | 1 | 1 | 11.76 |
| Lamiaceae | *Salvia pratensis* | Indicator | NA | NA | 1 | 1 | 47.06 |
| Lamiaceae | *Salvia verticillata* | Indicator | PD | NA | 1 | 1 | 23.53 |
| Lamiaceae | *Salvia vulgaris* | Indicator | NA | NA | 0 | 0 | 0.00 |
| Lamiaceae | *Satureja montana* | Indicator | PD | NA | 1 | 1 | 11.76 |
| Lamiaceae | *Sideritis endressii* | Indicator | NA | NA | 0 | 0 | 0.00 |
| Lamiaceae | *Sideritis glauca* | Protected | NA | 92/43/CEE | 0 | 0 | 0.00 |
| Lamiaceae | *Sideritis hyssopifolia* | Indicator | NA | NA | 0 | 0 | 0.00 |
| Lamiaceae | *Sideritis pungens* | Protected | NA | 92/43/CEE | 0 | 0 | 0.00 |
| Lamiaceae | *Stachys alopecuros* | Indicator | NA | NA | 0 | 0 | 0.00 |
| Lamiaceae | *Stachys officinalis* | Indicator | NA | NA | 1 | 1 | 23.53 |
| Lamiaceae | *Stachys recta* | Indicator | NA | NA | 1 | 1 | 11.76 |
| Lamiaceae | *Teucrium chamaedrys* | Indicator | PD | NA | 1 | 1 | 17.65 |
| Lamiaceae | *Teucrium montanum* | Indicator | PD | NA | 0 | 1 | 0.00 |
| Lamiaceae | *Teucrium pyrenaicum* | Indicator | NA | NA | 0 | 0 | 0.00 |
| Lamiaceae | *Thymus alpestris* | Indicator | NA | NA | 0 | 0 | 0.00 |
| Lamiaceae | *Thymus longicaulis* | Indicator | NA | NA | 0 | 0 | 0.00 |
| Lamiaceae | *Thymus nervosus* | Indicator | NA | NA | 0 | 0 | 0.00 |
| Lamiaceae | *Thymus odoratissimus* | Indicator | NA | NA | 1 | 0 | 5.88 |
| Lamiaceae | *Thymus praecox* | Indicator | NA | NA | 1 | 1 | 11.76 |
| Lamiaceae | *Thymus pulcherrimus* | Indicator | NA | NA | 0 | 0 | 0.00 |
| Lamiaceae | *Thymus pulegioides* | Indicator | ND | NA | 1 | 1 | 23.53 |
| Lamiaceae | *Thymus serpyllum* | Indicator | PD | NA | 1 | 1 | 11.76 |
| Lamiaceae | *Thymus striatus* | Indicator | NA | NA | 0 | 0 | 0.00 |
| Lamiaceae | *Thymus vulgaris* | Indicator | PD | NA | 1 | 1 | 17.65 |
| Leguminosae | *Anthyllis montana* | Indicator | NA | NA | 0 | 0 | 0.00 |
| Leguminosae | *Anthyllis vulneraria* | Indicator | PY | NA | 1 | 1 | 58.82 |
| Leguminosae | *Astragalus akkensis* | Indicator | NA | NA | 0 | 0 | 0.00 |
| Leguminosae | *Astragalus algarbiensis* | Protected | NA | 92/43/CEE | 0 | 0 | 0.00 |
| Leguminosae | *Astragalus alopecurus* | Protected | NA | 92/43/CEE | 0 | 1 | 0.00 |
| Leguminosae | *Astragalus aquilanus* | Protected | NA | 92/43/CEE | 0 | 0 | 0.00 |
| Leguminosae | *Astragalus danicus* | Indicator | NA | NA | 1 | 0 | 5.88 |
| Leguminosae | *Astragalus monspessulanus* | Indicator | PY | NA | 0 | 0 | 0.00 |
| Leguminosae | *Astragalus pelecinus* | Fodder | NA | 2014/362/EU | 1 | 0 | 5.88 |
| Leguminosae | *Astragalus sempervirens* | Indicator | NA | NA | 0 | 0 | 0.00 |
| Leguminosae | *Astragalus tremolsianus* | Protected | NA | 92/43/CEE | 0 | 0 | 0.00 |
| Leguminosae | *Astragalus verrucosus* | Protected | NA | 92/43/CEE | 0 | 1 | 0.00 |
| Leguminosae | *Chamaecytisus ratisbonensis* | Indicator | NA | NA | 1 | 0 | 5.88 |
| Leguminosae | *Coronilla minima* | Indicator | PY | NA | 0 | 1 | 0.00 |
| Leguminosae | *Cytisus hirsutus* | Indicator | NA | NA | 0 | 0 | 0.00 |
| Leguminosae | *Cytisus purgans* | Indicator | NA | NA | 0 | 0 | 0.00 |
| Leguminosae | *Dorycnium pentaphyllum* | Indicator | PY | NA | 1 | 1 | 5.88 |
| Leguminosae | *Genista anglica* | Indicator | PY | NA | 0 | 1 | 0.00 |
| Leguminosae | *Genista hispanica* | Indicator | PY | NA | 0 | 1 | 0.00 |
| Leguminosae | *Genista holopetala* | Protected | NA | 92/43/CEE | 0 | 0 | 0.00 |
| Leguminosae | *Genista pilosa* | Indicator | NA | NA | 0 | 1 | 0.00 |
| Leguminosae | *Genista radiata* | Indicator | NA | NA | 0 | 0 | 0.00 |
| Leguminosae | *Genista sagittalis* | Indicator | NA | NA | 1 | 0 | 5.88 |
| Leguminosae | *Genista scorpius* | Indicator | PY | NA | 1 | 1 | 5.88 |
| Leguminosae | *Genista tinctoria* | Indicator | NA | NA | 1 | 1 | 17.65 |
| Leguminosae | *Hedysarum hedysaroides* | Indicator | NA | NA | 0 | 0 | 0.00 |
| Leguminosae | *Hippocrepis comosa* | Indicator | PY | NA | 1 | 1 | 23.53 |
| Leguminosae | *Lathyrus linifolius* | Indicator | NA | NA | 0 | 0 | 0.00 |
| Leguminosae | *Lathyrus cicera* | Fodder | NA | 2014/362/EU | 0 | 0 | 0.00 |
| Leguminosae | *Lathyrus nissolia* | Indicator | PD | NA | 1 | 1 | 5.88 |
| Leguminosae | *Lathyrus pratensis* | Indicator | PY | NA | 1 | 1 | 41.18 |
| Leguminosae | *Lotus alpinus* | Indicator | PY | NA | 0 | 0 | 0.00 |
| Leguminosae | *Lotus corniculatus* | Fodder | PY | 66/401/EEC | 1 | 1 | 76.47 |
| Leguminosae | *Lotus pedunculatus* | Fodder | PY | 2014/362/EU | 1 | 1 | 35.29 |
| Leguminosae | *Lotus tenuis* | Fodder | PY | 2014/362/EU | 1 | 1 | 5.88 |
| Leguminosae | *Medicago doliata* | Fodder | NA | 2014/362/EU | 0 | 1 | 0.00 |
| Leguminosae | *Medicago littoralis* | Fodder | NA | 2014/362/EU | 0 | 1 | 0.00 |
| Leguminosae | *Medicago lupulina* | Fodder | PY | 66/401/EEC | 1 | 1 | 47.06 |
| Leguminosae | *Medicago murex* | Fodder | NA | 2014/362/EU | 0 | 1 | 0.00 |
| Leguminosae | *Medicago polymorpha* | Fodder | NA | 2014/362/EU | 1 | 1 | 5.88 |
| Leguminosae | *Medicago rugosa* | Fodder | NA | 2014/362/EU | 1 | 1 | 5.88 |
| Leguminosae | *Medicago sativa* | Fodder | PD | 66/401/EEC | 1 | 1 | 17.65 |
| Leguminosae | *Medicago sativa ssp. varia* | Fodder | NA | 66/401/EEC | 1 | 1 | 5.88 |
| Leguminosae | *Medicago scutellata* | Fodder | NA | 2014/362/EU | 1 | 1 | 5.88 |
| Leguminosae | *Medicago suffruticosa* | Indicator | NA | NA | 0 | 0 | 0.00 |
| Leguminosae | *Medicago tornata subsp. helix* | Fodder | NA | 2014/362/EU | 0 | 0 | 0.00 |
| Leguminosae | *Medicago truncatula* | Fodder | NA | 2014/362/EU | 1 | 1 | 5.88 |
| Leguminosae | *Onobrychis arenaria* | Indicator | NA | NA | 1 | 0 | 11.76 |
| Leguminosae | *Onobrychis conferta* | Indicator | NA | NA | 0 | 0 | 0.00 |
| Leguminosae | *Onobrychis supina* | Indicator | NA | NA | 0 | 0 | 0.00 |
| Leguminosae | *Onobrychis viciifolia* | Fodder | PY | 66/401/EEC | 1 | 1 | 29.41 |
| Leguminosae | *Ononis cristata* | Indicator | PY | NA | 0 | 1 | 0.00 |
| Leguminosae | *Ononis maweana* | Protected | NA | 92/43/CEE | 0 | 0 | 0.00 |
| Leguminosae | *Ononis spinosa* | Indicator | PY | NA | 1 | 1 | 35.29 |
| Leguminosae | *Ononis striata* | Indicator | NA | NA | 0 | 0 | 0.00 |
| Leguminosae | *Ornithopus compressus* | Fodder | NA | 2014/362/EU | 1 | 1 | 5.88 |
| Leguminosae | *Ornithopus sativus* | Fodder | NA | 2014/362/EU | 0 | 1 | 0.00 |
| Leguminosae | *Oxytropis campestris* | Indicator | PY | NA | 0 | 1 | 0.00 |
| Leguminosae | *Oxytropis carinthiaca* | Indicator | PY | NA | 0 | 0 | 0.00 |
| Leguminosae | *Oxytropis foucaudii* | Indicator | PY | NA | 0 | 0 | 0.00 |
| Leguminosae | *Oxytropis halleri* | Indicator | PY | NA | 0 | 1 | 0.00 |
| Leguminosae | *Oxytropis helvetica* | Indicator | NA | NA | 0 | 0 | 0.00 |
| Leguminosae | *Oxytropis jacquinii* | Indicator | PY | NA | 0 | 1 | 0.00 |
| Leguminosae | *Oxytropis neglecta* | Indicator | PY | NA | 0 | 0 | 0.00 |
| Leguminosae | *Pisum sativum* | Fodder | NA | 66/401/EEC | 0 | 1 | 0.00 |
| Leguminosae | *Securigera varia* | Indicator | PY | NA | 1 | 1 | 29.41 |
| Leguminosae | *Trifolium alpestre* | Indicator | NA | NA | 1 | 1 | 5.88 |
| Leguminosae | *Trifolium alpinum* | Indicator | PY | NA | 0 | 1 | 0.00 |
| Leguminosae | *Trifolium badium* | Indicator | NA | NA | 0 | 1 | 0.00 |
| Leguminosae | *Trifolium campestre* | Indicator | PY | NA | 1 | 1 | 17.65 |
| Leguminosae | *Trifolium dubium* | Indicator | PY | NA | 1 | 1 | 11.76 |
| Leguminosae | *Trifolium fragiferum* | Fodder | NA | 2014/362/EU | 1 | 1 | 11.76 |
| Leguminosae | *Trifolium glanduliferum* | Fodder | NA | 2014/362/EU | 1 | 1 | 5.88 |
| Leguminosae | *Trifolium hirtum* | Fodder | NA | 2014/362/EU | 0 | 1 | 0.00 |
| Leguminosae | *Trifolium hybridum* | Fodder | NA | 66/401/EEC | 0 | 1 | 0.00 |
| Leguminosae | *Trifolium incarnatum* | Fodder | NA | 66/401/EEC | 1 | 1 | 5.88 |
| Leguminosae | *Trifolium isthmocarpum* | Fodder | PY | 2014/362/EU | 0 | 1 | 0.00 |
| Leguminosae | *Trifolium michelianum* | Fodder | NA | 2014/362/EU | 0 | 0 | 0.00 |
| Leguminosae | *Trifolium montanum* | Indicator | PY | NA | 1 | 1 | 23.53 |
| Leguminosae | *Trifolium ochroleucon* | Indicator | NA | NA | 1 | 1 | 11.76 |
| Leguminosae | *Trifolium pratense* | Fodder | PY | 66/401/EEC | 1 | 1 | 47.06 |
| Leguminosae | *Trifolium repens* | Fodder | PY | 66/401/EEC | 1 | 1 | 29.41 |
| Leguminosae | *Trifolium squamosum* | Indicator | NA | NA | 0 | 1 | 0.00 |
| Leguminosae | *Trifolium squarrosum* | Fodder | NA | 2014/362/EU | 0 | 0 | 0.00 |
| Leguminosae | *Trifolium subterraneum* | Fodder | NA | 2014/362/EU | 1 | 1 | 5.88 |
| Leguminosae | *Trifolium thalii* | Indicator | PY | NA | 0 | 1 | 0.00 |
| Leguminosae | *Trifolium vesiculosum* | Fodder | NA | 2014/362/EU | 1 | 1 | 5.88 |
| Leguminosae | *Vicia sativa* | Fodder | NA | 66/401/EEC | 0 | 0 | 0.00 |
| Leguminosae | *Vicia benghalensis* | Fodder | PY | 2014/362/EU | 0 | 1 | 0.00 |
| Leguminosae | *Vicia cracca* | Fodder | PY | 66/401/EEC | 1 | 1 | 41.18 |
| Leguminosae | *Vicia faba* | Fodder | NA | 66/401/EEC | 1 | 1 | 5.88 |
| Leguminosae | *Vicia pannonica* | Fodder | NA | 66/401/EEC | 0 | 1 | 0.00 |
| Leguminosae | *Vicia pyrenaica* | Fodder | NA | 66/401/EEC | 0 | 0 | 0.00 |
| Leguminosae | *Vicia sativa* | Fodder | PY | 66/401/EEC | 1 | 1 | 23.53 |
| Leguminosae | *Vicia sepium* | Fodder | NA | 66/401/EEC | 1 | 1 | 29.41 |
| Leguminosae | *Vicia villosa* | Fodder | NA | 66/401/EEC | 1 | 1 | 5.88 |
| Lentibulariaceae | *Pinguicula alpina* | Indicator | NA | NA | 0 | 0 | 0.00 |
| Lentibulariaceae | *Pinguicula nevadensis* | Protected | NA | 92/43/CEE | 0 | 0 | 0.00 |
| Liliaceae | *Erythronium dens-canis* | Indicator | NA | NA | 0 | 1 | 0.00 |
| Liliaceae | *Fritillaria drenovskii* | Protected | NA | 92/43/CEE | 0 | 0 | 0.00 |
| Liliaceae | *Fritillaria gussichiae* | Protected | NA | 92/43/CEE | 0 | 0 | 0.00 |
| Liliaceae | *Fritillaria pyrenaica* | Indicator | NA | NA | 0 | 0 | 0.00 |
| Liliaceae | *Gagea serotina* | Indicator | NA | NA | 0 | 0 | 0.00 |
| Liliaceae | *Lilium bulbiferum* | Indicator | NA | NA | 1 | 0 | 5.88 |
| Liliaceae | *Lilium martagon* | Indicator | MPD | NA | 1 | 1 | 17.65 |
| Linaceae | *Linum bienne* | Indicator | NA | NA | 1 | 0 | 11.76 |
| Linaceae | *Linum capitatum* | Indicator | NA | NA | 0 | 0 | 0.00 |
| Linaceae | *Linum catharticum* | Indicator | PD | NA | 1 | 1 | 5.88 |
| Linaceae | *Linum narbonense* | Indicator | NA | NA | 0 | 1 | 0.00 |
| Linaceae | *Linum perenne* | Indicator | NA | NA | 1 | 1 | 5.88 |
| Linaceae | *Linum tenuifolium* | Indicator | NA | NA | 1 | 1 | 11.76 |
| Linaceae | *Linum usitatissimum* | Indicator | ND | NA | 0 | 1 | 0.00 |
| Linaceae | *Linum viscosum* | Indicator | NA | NA | 0 | 0 | 0.00 |
| Malvaceae | *Kosteletzkya pentacarpos* | Protected | NA | 92/43/CEE | 0 | 0 | 0.00 |
| Malvaceae | *Malva moschata* | Indicator | NA | NA | 1 | 0 | 52.94 |
| Malvaceae | *Malva sylvestris* | Indicator | NA | NA | 1 | 0 | 52.94 |
| Melanthiaceae | *Veratrum album* | Indicator | NA | NA | 0 | 0 | 0.00 |
| Melanthiaceae | *Veratrum lobelianum* | Indicator | NA | NA | 0 | 0 | 0.00 |
| Onagraceae | *Epilobium anagallidifolium* | Indicator | NA | NA | 0 | 1 | 0.00 |
| Onagraceae | *Epilobium angustifolium* | Indicator | PD | NA | 1 | 1 | 35.29 |
| Orchidaceae | *Anacamptis morio* | Indicator | NA | NA | 0 | 0 | 0.00 |
| Orchidaceae | *Cypripedium calceolus* | Protected | NA | 92/43/CEE | 0 | 1 | 0.00 |
| Orchidaceae | *Dactylorhiza maculata* | Indicator | NA | NA | 0 | 1 | 0.00 |
| Orchidaceae | *Dactylorhiza sambucina* | Indicator | NA | NA | 0 | 0 | 0.00 |
| Orchidaceae | *Dactylorhiza viridis* | Indicator | NA | NA | 0 | 1 | 0.00 |
| Orchidaceae | *Gymnadenia conopsea* | Indicator | NA | NA | 1 | 1 | 5.88 |
| Orchidaceae | *Gymnadenia nigra* | Indicator | NA | NA | 0 | 0 | 0.00 |
| Orchidaceae | *Liparis loeselii* | Protected | NA | 92/43/CEE | 0 | 0 | 0.00 |
| Orchidaceae | *Neotinea ustulata* | Indicator | NA | NA | 0 | 0 | 0.00 |
| Orchidaceae | *Ophrys argolica* | Protected | NA | 92/43/CEE | 0 | 0 | 0.00 |
| Orchidaceae | *Ophrys lunulata* | Protected | NA | 92/43/CEE | 0 | 0 | 0.00 |
| Orchidaceae | *Orchis mascula* | Indicator | NA | NA | 0 | 0 | 0.00 |
| Orchidaceae | *Plantanthera bifolia* | Indicator | NA | NA | 0 | 0 | 0.00 |
| Orchidaceae | *Pseudorchis albida* | Indicator | NA | NA | 0 | 0 | 0.00 |
| Orchidaceae | *Serapias cordigera* | Indicator | NA | NA | 0 | 0 | 0.00 |
| Orchidaceae | *Spiranthes aestivalis* | Protected | NA | 92/43/CEE | 0 | 0 | 0.00 |
| Orchidaceae | *Traunsteinera globosa* | Indicator | NA | NA | 0 | 0 | 0.00 |
| Orobanchaceae | *Bartsia alpina* | Indicator | PD | NA | 0 | 1 | 0.00 |
| Orobanchaceae | *Euphrasia frigida* | Indicator | NA | NA | 0 | 1 | 0.00 |
| Orobanchaceae | *Euphrasia genargentea* | Protected | NA | 92/43/CEE | 0 | 0 | 0.00 |
| Orobanchaceae | *Euphrasia hirtella* | Indicator | PD | NA | 0 | 1 | 0.00 |
| Orobanchaceae | *Euphrasia marchesettii* | Protected | NA | 92/43/CEE | 0 | 0 | 0.00 |
| Orobanchaceae | *Euphrasia minima* | Indicator | PD | NA | 0 | 1 | 0.00 |
| Orobanchaceae | *Euphrasia nemorosa* | Indicator | NA | NA | 0 | 1 | 0.00 |
| Orobanchaceae | *Euphrasia officinalis* | Indicator | NA | NA | 1 | 0 | 17.65 |
| Orobanchaceae | *Euphrasia picta* | Indicator | NA | NA | 0 | 0 | 0.00 |
| Orobanchaceae | *Euphrasia rostkoviana* | Indicator | NA | NA | 1 | 1 | 5.88 |
| Orobanchaceae | *Euphrasia salisburgensis* | Indicator | NA | NA | 0 | 1 | 0.00 |
| Orobanchaceae | *Euphrasia stricta* | Indicator | NA | NA | 0 | 0 | 0.00 |
| Orobanchaceae | *Melampyrum pratense* | Indicator | PD | NA | 1 | 1 | 5.88 |
| Orobanchaceae | *Melampyrum sylvaticum* | Indicator | PD | NA | 0 | 1 | 0.00 |
| Orobanchaceae | *Orthantha lutea* | Indicator | NA | NA | 0 | 0 | 0.00 |
| Orobanchaceae | *Pedicularis ascendens* | Indicator | NA | NA | 0 | 0 | 0.00 |
| Orobanchaceae | *Pedicularis cenisia* | Indicator | NA | NA | 0 | 0 | 0.00 |
| Orobanchaceae | *Pedicularis comosa* | Indicator | NA | NA | 0 | 0 | 0.00 |
| Orobanchaceae | *Pedicularis elegans* | Indicator | NA | NA | 0 | 0 | 0.00 |
| Orobanchaceae | *Pedicularis foliosa* | Indicator | NA | NA | 0 | 0 | 0.00 |
| Orobanchaceae | *Pedicularis kerneri* | Indicator | NA | NA | 0 | 0 | 0.00 |
| Orobanchaceae | *Pedicularis oederi* | Indicator | PD | NA | 0 | 1 | 0.00 |
| Orobanchaceae | *Pedicularis pyrenaica* | Indicator | PD | NA | 0 | 0 | 0.00 |
| Orobanchaceae | *Pedicularis rostratocapitata* | Indicator | NA | NA | 0 | 0 | 0.00 |
| Orobanchaceae | *Pedicularis sylvatica* | Indicator | NA | NA | 0 | 1 | 0.00 |
| Orobanchaceae | *Pedicularis tuberosa* | Indicator | PD | NA | 0 | 0 | 0.00 |
| Orobanchaceae | *Pedicularis verticillata* | Indicator | PD | NA | 0 | 1 | 0.00 |
| Orobanchaceae | *Rhinanthus alectorolophus* | Indicator | PD | NA | 1 | 1 | 23.53 |
| Orobanchaceae | *Rhinanthus aristatus* | Indicator | PD | NA | 0 | 1 | 0.00 |
| Orobanchaceae | *Rhinanthus glacialis* | Indicator | PD | NA | 0 | 1 | 0.00 |
| Orobanchaceae | *Rhinanthus mediterraneus* | Indicator | PD | NA | 0 | 1 | 0.00 |
| Orobanchaceae | *Rhinanthus minor* | Indicator | PD | NA | 1 | 1 | 29.41 |
| Orobanchaceae | *Rhinanthus wettsteinii* | Indicator | NA | NA | 0 | 0 | 0.00 |
| Oxalidaceae | *Oxalis acetosella* | Indicator | PD | NA | 0 | 1 | 0.00 |
| Paeoniaceae | *Paeonia cambessedesii* | Protected | NA | 92/43/CEE | 0 | 0 | 0.00 |
| Paeoniaceae | *Paeonia parnassica* | Protected | NA | 92/43/CEE | 0 | 0 | 0.00 |
| Papaveraceae | *Papaver rhoeas* | Indicator | ND | NA | 1 | 1 | 82.35 |
| Plantaginaceae | *Digitalis ferruginea* | Indicator | NA | NA | 1 | 1 | 5.88 |
| Plantaginaceae | *Digitalis grandiflora* | Indicator | NA | NA | 1 | 1 | 11.76 |
| Plantaginaceae | *Erinus alpinus* | Indicator | NA | NA | 1 | 1 | 5.88 |
| Plantaginaceae | *Globularia cordifolia* | Indicator | NA | NA | 0 | 1 | 0.00 |
| Plantaginaceae | *Globularia meridionalis* | Indicator | NA | NA | 0 | 0 | 0.00 |
| Plantaginaceae | *Globularia nudicaulis* | Indicator | NA | NA | 0 | 0 | 0.00 |
| Plantaginaceae | *Globularia punctata* | Indicator | MPD | NA | 1 | 1 | 5.88 |
| Plantaginaceae | *Globularia repens* | Indicator | NA | NA | 0 | 0 | 0.00 |
| Plantaginaceae | *Linaria algarviana* | Protected | NA | 92/43/CEE | 0 | 0 | 0.00 |
| Plantaginaceae | *Linaria alpina* | Indicator | PD | NA | 0 | 1 | 0.00 |
| Plantaginaceae | *Linaria coutinhoi* | Protected | NA | 92/43/CEE | 0 | 0 | 0.00 |
| Plantaginaceae | *Linaria flava* | Protected | NA | 92/43/CEE | 0 | 0 | 0.00 |
| Plantaginaceae | *Linaria hellenica* | Protected | NA | 92/43/CEE | 0 | 0 | 0.00 |
| Plantaginaceae | *Linaria purpurea* | Indicator | PD | NA | 1 | 1 | 11.76 |
| Plantaginaceae | *Linaria tursica* | Protected | NA | 92/43/CEE | 0 | 0 | 0.00 |
| Plantaginaceae | *Plantago alpina* | Indicator | NA | NA | 1 | 1 | 5.88 |
| Plantaginaceae | *Plantago atrata* | Indicator | NA | NA | 1 | 1 | 5.88 |
| Plantaginaceae | *Plantago lanceolata* | Fodder | PD | 2014/362/EU | 1 | 1 | 70.59 |
| Plantaginaceae | *Plantago major* | Indicator | PD | NA | 1 | 1 | 23.53 |
| Plantaginaceae | *Plantago maritima* | Indicator | PD | NA | 1 | 1 | 17.65 |
| Plantaginaceae | *Plantago media* | Indicator | PD | NA | 1 | 1 | 29.41 |
| Plantaginaceae | *Plantago monosperma* | Indicator | NA | NA | 0 | 0 | 0.00 |
| Plantaginaceae | *Plantago subulata* | Indicator | NA | NA | 0 | 0 | 0.00 |
| Plantaginaceae | *Veronica bellidioides* | Indicator | NA | NA | 0 | 0 | 0.00 |
| Plantaginaceae | *Veronica filiformis* | Indicator | NA | NA | 0 | 0 | 0.00 |
| Plantaginaceae | *Veronica allionii* | Indicator | NA | NA | 0 | 0 | 0.00 |
| Plantaginaceae | *Veronica alpina* | Indicator | PD | NA | 1 | 1 | 5.88 |
| Plantaginaceae | *Veronica arvensis* | Indicator | PD | NA | 0 | 1 | 0.00 |
| Plantaginaceae | *Veronica austriaca* | Indicator | NA | NA | 0 | 0 | 0.00 |
| Plantaginaceae | *Veronica bellidioides* | Indicator | NA | NA | 0 | 1 | 0.00 |
| Plantaginaceae | *Veronica chamaedrys* | Indicator | NA | NA | 1 | 1 | 35.29 |
| Plantaginaceae | *Veronica fruticans* | Indicator | PD | NA | 0 | 1 | 0.00 |
| Plantaginaceae | *Veronica micrantha* | Protected | NA | 92/43/CEE | 0 | 0 | 0.00 |
| Plantaginaceae | *Veronica officinalis* | Indicator | PD | NA | 1 | 1 | 23.53 |
| Plantaginaceae | *Veronica persica* | Indicator | NA | NA | 1 | 1 | 11.76 |
| Plantaginaceae | *Veronica ponae* | Indicator | NA | NA | 0 | 0 | 0.00 |
| Plantaginaceae | *Veronica serpyllifolia* | Indicator | NA | NA | 1 | 1 | 5.88 |
| Plantaginaceae | *Veronica spicata* | Indicator | PD | NA | 1 | 1 | 23.53 |
| Plantaginaceae | *Veronica verna* | Indicator | NA | NA | 0 | 1 | 0.00 |
| Plumbaginaceae | *Armeria alpina* | Indicator | NA | NA | 0 | 0 | 0.00 |
| Plumbaginaceae | *Armeria arenaria* | Indicator | NA | NA | 0 | 1 | 0.00 |
| Plumbaginaceae | *Armeria canescens* | Indicator | NA | NA | 0 | 0 | 0.00 |
| Plumbaginaceae | *Armeria cantabrica* | Indicator | PD | NA | 0 | 0 | 0.00 |
| Plumbaginaceae | *Armeria majellensis* | Indicator | NA | NA | 0 | 0 | 0.00 |
| Plumbaginaceae | *Armeria maritima* | Indicator | PD | NA | 1 | 1 | 35.29 |
| Plumbaginaceae | *Armeria pubinervis* | Indicator | NA | NA | 0 | 0 | 0.00 |
| Plumbaginaceae | *Armeria velutina* | Protected | NA | 92/43/CEE | 0 | 0 | 0.00 |
| Plumbaginaceae | *Limonium lanceolatum* | Protected | NA | 92/43/CEE | 0 | 0 | 0.00 |
| Plumbaginaceae | *Limonium minutum* | Indicator | NA | NA | 0 | 0 | 0.00 |
| Poaceae | *Agrostis alpina* | Fodder | NA | 66/401/EEC | 0 | 0 | 0.00 |
| Poaceae | *Agrostis canina* | Fodder | NA | 66/401/EEC | 1 | 1 | 5.88 |
| Poaceae | *Agrostis capillaris* | Fodder | NA | 66/401/EEC | 1 | 1 | 17.65 |
| Poaceae | *Agrostis castellana* | Fodder | NA | 66/401/EEC | 0 | 1 | 0.00 |
| Poaceae | *Agrostis curtisii* | Fodder | ND | 66/401/EEC | 1 | 1 | 5.88 |
| Poaceae | *Agrostis delicatula* | Fodder | NA | 66/401/EEC | 0 | 1 | 0.00 |
| Poaceae | *Agrostis nevadensis* | Fodder | NA | 66/401/EEC | 0 | 0 | 0.00 |
| Poaceae | *Agrostis rupestris* | Fodder | NA | 66/401/EEC | 1 | 0 | 5.88 |
| Poaceae | *Agrostis schleicheri* | Fodder | NA | 66/401/EEC | 0 | 0 | 0.00 |
| Poaceae | *Agrostis stolonifera* | Fodder | NA | 66/401/EEC | 1 | 1 | 11.76 |
| Poaceae | *Aira caryophyllea* | Indicator | NA | NA | 1 | 1 | 5.88 |
| Poaceae | *Alopecurus myosuroides* | Fodder | NA | 66/401/EEC | 1 | 1 | 5.88 |
| Poaceae | *Alopecurus pratensis* | Fodder | PD | 66/401/EEC | 1 | 1 | 23.53 |
| Poaceae | *Alopecurus utriculatus* | Indicator | NA | NA | 0 | 0 | 0.00 |
| Poaceae | *Anthoxanthum aristatum* | Indicator | NA | NA | 0 | 0 | 0.00 |
| Poaceae | *Anthoxanthum odoratum* | Indicator | PD | NA | 1 | 1 | 35.29 |
| Poaceae | *Arrhenatherum elatius* | Fodder | NA | 66/401/EEC | 1 | 1 | 17.65 |
| Poaceae | *Arrhenatherum longifolium* | Indicator | NA | NA | 0 | 0 | 0.00 |
| Poaceae | *Avena fatua* | Fodder | NA | 66/401/EEC | 1 | 1 | 5.88 |
| Poaceae | *Avenula mirandana* | Indicator | NA | NA | 0 | 0 | 0.00 |
| Poaceae | *Bothriochloa ischaemum* | Indicator | NA | NA | 1 | 1 | 5.88 |
| Poaceae | *Brachypodium phoenicoides* | Indicator | NA | NA | 1 | 0 | 5.88 |
| Poaceae | *Brachypodium pinnatum* | Indicator | PD | NA | 1 | 1 | 17.65 |
| Poaceae | *Brachypodium sylvaticum* | Indicator | NA | NA | 1 | 1 | 11.76 |
| Poaceae | *Briza media* | Indicator | PD | NA | 1 | 1 | 47.06 |
| Poaceae | *Briza minor* | Indicator | NA | NA | 0 | 1 | 0.00 |
| Poaceae | *Bromus erectus* | Indicator | ND | NA | 1 | 1 | 17.65 |
| Poaceae | *Bromus hordeaceus* | Indicator | PD | NA | 0 | 1 | 0.00 |
| Poaceae | *Calamagrostis arundinacea* | Indicator | NA | NA | 0 | 1 | 0.00 |
| Poaceae | *Calamagrostis varia* | Indicator | NA | NA | 1 | 0 | 5.88 |
| Poaceae | *Coleanthus subtilis* | Protected | NA | 92/43/CEE | 0 | 0 | 0.00 |
| Poaceae | *Cynosurus cristatus* | Indicator | NA | NA | 1 | 1 | 23.53 |
| Poaceae | *Dactylis glomerata* | Fodder | PD | 66/401/EEC | 1 | 1 | 23.53 |
| Poaceae | *Danthonia decumbens* | Indicator | PD | NA | 1 | 1 | 5.88 |
| Poaceae | *Deschampsia cespitosa* | Indicator | PD | NA | 1 | 1 | 17.65 |
| Poaceae | *Deschampsia flexuosa* | Indicator | PD | NA | 1 | 1 | 23.53 |
| Poaceae | *Elymus repens* | Indicator | PD | NA | 0 | 1 | 0.00 |
| Poaceae | *Festuca airoides* | Indicator | PD | NA | 0 | 0 | 0.00 |
| Poaceae | *Festuca arundinacea* | Fodder | NA | 66/401/EEC | 1 | 1 | 17.65 |
| Poaceae | *Festuca brigantina* | Protected | NA | 92/43/CEE | 0 | 0 | 0.00 |
| Poaceae | *Festuca circummediterranea* | Indicator | NA | NA | 0 | 0 | 0.00 |
| Poaceae | *Festuca dalmatica* | Indicator | NA | NA | 0 | 0 | 0.00 |
| Poaceae | *Festuca duriotagana* | Protected | NA | 92/43/CEE | 0 | 0 | 0.00 |
| Poaceae | *Festuca elegans* | Protected | NA | 92/43/CEE | 0 | 0 | 0.00 |
| Poaceae | *Festuca eskia* | Indicator | NA | NA | 0 | 0 | 0.00 |
| Poaceae | *Festuca filiformis* | Indicator | NA | NA | 1 | 1 | 5.88 |
| Poaceae | *Festuca gautieri* | Indicator | NA | NA | 0 | 0 | 0.00 |
| Poaceae | *Festuca glacialis* | Indicator | NA | NA | 0 | 0 | 0.00 |
| Poaceae | *Festuca halleri* | Indicator | PD | NA | 0 | 1 | 0.00 |
| Poaceae | *Festuca henriquesii* | Indicator | NA | 92/43/CEE | 0 | 0 | 0.00 |
| Poaceae | *Festuca iberica* | Indicator | NA | NA | 0 | 0 | 0.00 |
| Poaceae | *Festuca inops* | Indicator | NA | NA | 0 | 0 | 0.00 |
| Poaceae | *Festuca nigrescens* | Indicator | NA | NA | 1 | 1 | 5.88 |
| Poaceae | *Festuca norica* | Indicator | NA | NA | 0 | 0 | 0.00 |
| Poaceae | *Festuca ovina* | Fodder | PD | 66/401/EEC | 1 | 1 | 23.53 |
| Poaceae | *Festuca pallens* | Indicator | NA | NA | 1 | 0 | 5.88 |
| Poaceae | *Festuca paniculata* | Indicator | NA | NA | 0 | 1 | 0.00 |
| Poaceae | *Festuca pratensis* | Fodder | PD | 66/401/EEC | 1 | 1 | 11.76 |
| Poaceae | *Festuca pseudodalmatica* | Indicator | NA | NA | 0 | 0 | 0.00 |
| Poaceae | *Festuca pseudodura* | Indicator | NA | NA | 0 | 0 | 0.00 |
| Poaceae | *Festuca quadriflora* | Indicator | NA | NA | 0 | 0 | 0.00 |
| Poaceae | *Festuca rivularis* | Indicator | NA | NA | 0 | 0 | 0.00 |
| Poaceae | *Festuca rothmaleri* | Indicator | NA | NA | 0 | 0 | 0.00 |
| Poaceae | *Festuca rubra* | Fodder | PD | 66/401/EEC | 1 | 1 | 11.76 |
| Poaceae | *Festuca rupicaprina* | Indicator | NA | NA | 1 | 0 | 5.88 |
| Poaceae | *Festuca rupicola* | Indicator | NA | NA | 1 | 1 | 5.88 |
| Poaceae | *Festuca scabriculmis* | Indicator | PD | NA | 0 | 0 | 0.00 |
| Poaceae | *Festuca summilusitana* | Protected | NA | 92/43/CEE | 0 | 0 | 0.00 |
| Poaceae | *Festuca valesiaca* | Indicator | NA | NA | 1 | 1 | 11.76 |
| Poaceae | *Festuca varia* | Indicator | NA | NA | 0 | 1 | 0.00 |
| Poaceae | *Festuca violacea* | Indicator | NA | NA | 0 | 0 | 0.00 |
| Poaceae | *Festuca vivipara* | Indicator | NA | NA | 0 | 1 | 0.00 |
| Poaceae | *Gaudinia hispanica* | Protected | NA | 92/43/CEE | 0 | 0 | 0.00 |
| Poaceae | *Helictochloa versicolor* | Indicator | NA | NA | 0 | 0 | 0.00 |
| Poaceae | *Helictotrichon cantabricum* | Indicator | NA | NA | 0 | 0 | 0.00 |
| Poaceae | *Helictotrichon desertorum* | Indicator | NA | NA | 0 | 0 | 0.00 |
| Poaceae | *Helictotrichon marginatum* | Indicator | NA | NA | 0 | 0 | 0.00 |
| Poaceae | *Helictotrichon planiculme* | Indicator | NA | NA | 0 | 0 | 0.00 |
| Poaceae | *Helictotrichon pratense* | Indicator | NA | NA | 0 | 1 | 0.00 |
| Poaceae | *Helictotrichon pubescens* | Indicator | NA | NA | 1 | 1 | 11.76 |
| Poaceae | *Helictotrichon sedenense* | Indicator | PD | NA | 0 | 0 | 0.00 |
| Poaceae | *Helictotrichon versicolor* | Indicator | PD | NA | 0 | 1 | 0.00 |
| Poaceae | *Holcus annuus* | Protected | NA | 92/43/CEE | 0 | 0 | 0.00 |
| Poaceae | *Holcus lanatus* | Indicator | ND | NA | 1 | 1 | 23.53 |
| Poaceae | *Holcus mollis* | Indicator | NA | NA | 1 | 1 | 5.88 |
| Poaceae | *Hordeum secalinum* | Indicator | NA | NA | 0 | 0 | 0.00 |
| Poaceae | *Koeleria brevis* | Indicator | NA | NA | 0 | 0 | 0.00 |
| Poaceae | *Koeleria eriostachya* | Indicator | NA | NA | 0 | 0 | 0.00 |
| Poaceae | *Koeleria macrantha* | Indicator | PD | NA | 1 | 1 | 5.88 |
| Poaceae | *Koeleria pyramidata* | Indicator | NA | NA | 1 | 0 | 11.76 |
| Poaceae | *Koeleria splendens* | Indicator | PY | NA | 0 | 0 | 0.00 |
| Poaceae | *Koeleria vallesiana* | Indicator | NA | NA | 0 | 1 | 0.00 |
| Poaceae | *Lolium multiflorum* | Fodder | PD | 66/401/EEC | 1 | 1 | 5.88 |
| Poaceae | *Lolium perenne* | Fodder | PY | 66/401/EEC | 1 | 1 | 23.53 |
| Poaceae | *Melica ciliata* | Indicator | ND | NA | 1 | 1 | 5.88 |
| Poaceae | *Molinia caerulea* | Indicator | NA | NA | 1 | 1 | 29.41 |
| Poaceae | *Nardus stricta* | Indicator | PD | NA | 1 | 1 | 11.76 |
| Poaceae | *Oreochloa disticha* | Indicator | NA | NA | 0 | 0 | 0.00 |
| Poaceae | *Phleum alpinum* | Indicator | ND | NA | 1 | 1 | 11.76 |
| Poaceae | *Phleum hirsutum* | Indicator | NA | NA | 0 | 1 | 0.00 |
| Poaceae | *Phleum phleoides* | Indicator | NA | NA | 1 | 1 | 11.76 |
| Poaceae | *Phleum pratense* | Fodder | NA | 66/401/EEC | 1 | 1 | 11.76 |
| Poaceae | *Poa alpina* | Fodder | PD | 66/401/EEC | 1 | 1 | 11.76 |
| Poaceae | *Poa angustifolia* | Fodder | NA | 66/401/EEC | 1 | 1 | 5.88 |
| Poaceae | *Poa annua* | Fodder | NA | 66/401/EEC | 1 | 1 | 5.88 |
| Poaceae | *Poa badensis* | Fodder | NA | 66/401/EEC | 0 | 0 | 0.00 |
| Poaceae | *Poa bulbosa* | Fodder | NA | 66/401/EEC | 1 | 1 | 5.88 |
| Poaceae | *Poa chaixii* | Fodder | NA | 66/401/EEC | 0 | 0 | 0.00 |
| Poaceae | *Poa compressa* | Fodder | NA | 66/401/EEC | 1 | 1 | 11.76 |
| Poaceae | *Poa nemoralis* | Fodder | NA | 66/401/EEC | 1 | 1 | 17.65 |
| Poaceae | *Poa pratensis* | Fodder | PD | 66/401/EEC | 1 | 1 | 29.41 |
| Poaceae | *Poa supina* | Fodder | NA | 66/401/EEC | 1 | 0 | 11.76 |
| Poaceae | *Poa trivialis* | Fodder | ND | 66/401/EEC | 1 | 1 | 5.88 |
| Poaceae | *Poa variegata* | Fodder | NA | 66/401/EEC | 0 | 0 | 0.00 |
| Poaceae | *Puccinellia fasciculata* | Protected | NA | 92/43/CEE | 0 | 1 | 0.00 |
| Poaceae | *Sesleria albicans* | Indicator | PD | NA | 0 | 1 | 0.00 |
| Poaceae | *Sesleria caerulea* | Indicator | ND | NA | 1 | 1 | 11.76 |
| Poaceae | *Sesleria coerulans* | Indicator | NA | NA | 0 | 0 | 0.00 |
| Poaceae | *Sesleria sadleriana* | Indicator | NA | NA | 0 | 1 | 0.00 |
| Poaceae | *Sesleria sphaerocephala* | Indicator | ND | NA | 0 | 0 | 0.00 |
| Poaceae | *Sesleria tenuifolia* | Indicator | NA | NA | 0 | 0 | 0.00 |
| Poaceae | *Stipa austroitalica* | Protected | NA | 92/43/CEE | 0 | 0 | 0.00 |
| Poaceae | *Stipa pennata* | Indicator | NA | NA | 0 | 1 | 0.00 |
| Poaceae | *Trisetum alpestre* | Indicator | NA | NA | 0 | 0 | 0.00 |
| Poaceae | *Trisetum flavescens* | Fodder | PD | 66/401/EEC | 1 | 1 | 17.65 |
| Polygalaceae | *Polygala alpestris* | Indicator | NA | NA | 0 | 0 | 0.00 |
| Polygalaceae | *Polygala alpina* | Indicator | PD | NA | 0 | 0 | 0.00 |
| Polygalaceae | *Polygala calcarea* | Indicator | NA | NA | 0 | 0 | 0.00 |
| Polygalaceae | *Polygala chamaebuxus* | Indicator | NA | NA | 1 | 0 | 5.88 |
| Polygalaceae | *Polygala comosa* | Indicator | NA | NA | 1 | 1 | 5.88 |
| Polygalaceae | *Polygala major* | Indicator | NA | NA | 1 | 0 | 5.88 |
| Polygalaceae | *Polygala multicaulis* | Indicator | NA | NA | 0 | 0 | 0.00 |
| Polygalaceae | *Polygala serpyllifolia* | Indicator | NA | NA | 0 | 0 | 0.00 |
| Polygalaceae | *Polygala vulgaris* | Indicator | NA | NA | 1 | 1 | 5.88 |
| Polygonaceae | *Persicaria bistorta* | Indicator | NA | NA | 1 | 1 | 11.76 |
| Polygonaceae | *Persicaria vivipara* | Indicator | PD | NA | 0 | 0 | 0.00 |
| Polygonaceae | *Polygonum aviculare* | Indicator | PD | NA | 1 | 1 | 5.88 |
| Polygonaceae | *Rumex acetosa* | Indicator | PD | NA | 1 | 1 | 52.94 |
| Polygonaceae | *Rumex acetosella* | Indicator | NA | NA | 1 | 1 | 35.29 |
| Polygonaceae | *Rumex alpestris* | Indicator | NA | NA | 0 | 0 | 0.00 |
| Polygonaceae | *Rumex crispus* | Indicator | PD | NA | 1 | 1 | 17.65 |
| Polygonaceae | *Rumex nebroides* | Indicator | NA | NA | 0 | 0 | 0.00 |
| Polygonaceae | *Rumex obtusifolius* | Indicator | ND | NA | 0 | 1 | 0.00 |
| Polygonaceae | *Rumex scutatus* | Indicator | PD | NA | 1 | 1 | 5.88 |
| Primulaceae | *Androsace chamaejasme* | Indicator | NA | NA | 0 | 1 | 0.00 |
| Primulaceae | *Androsace laggeri* | Indicator | NA | NA | 0 | 0 | 0.00 |
| Primulaceae | *Androsace obtusifolia* | Indicator | NA | NA | 0 | 0 | 0.00 |
| Primulaceae | *Androsace villosa* | Indicator | ND | NA | 0 | 0 | 0.00 |
| Primulaceae | *Lysimachia nummularia* | Indicator | NA | NA | 1 | 0 | 11.76 |
| Primulaceae | *Primula auricula* | Indicator | NA | NA | 1 | 1 | 5.88 |
| Primulaceae | *Primula clusiana* | Indicator | NA | NA | 0 | 0 | 0.00 |
| Primulaceae | *Primula elatior* | Indicator | ND | NA | 1 | 1 | 35.29 |
| Primulaceae | *Primula farinosa* | Indicator | NA | NA | 0 | 1 | 0.00 |
| Primulaceae | *Primula glutinosa* | Indicator | NA | NA | 0 | 0 | 0.00 |
| Primulaceae | *Primula hirsuta* | Indicator | ND | NA | 0 | 0 | 0.00 |
| Primulaceae | *Primula integrifolia* | Indicator | NA | NA | 0 | 0 | 0.00 |
| Primulaceae | *Primula minima* | Indicator | NA | NA | 0 | 0 | 0.00 |
| Primulaceae | *Primula veris* | Indicator | PD | NA | 1 | 1 | 70.59 |
| Primulaceae | *Primula vulgaris* | Indicator | NA | NA | 1 | 1 | 17.65 |
| Primulaceae | *Soldanella alpina* | Indicator | ND | NA | 0 | 1 | 0.00 |
| Primulaceae | *Soldanella carpatica* | Indicator | NA | NA | 0 | 0 | 0.00 |
| Primulaceae | *Soldanella pusilla* | Indicator | PD | NA | 0 | 1 | 0.00 |
| Primulaceae | *Vitaliana primuliflora* | Indicator | NA | NA | 0 | 0 | 0.00 |
| Ranunculaceae | *Aconitum anthora* | Indicator | NA | NA | 1 | 0 | 5.88 |
| Ranunculaceae | *Aconitum lycoctonum* | Indicator | MPD | NA | 1 | 1 | 11.76 |
| Ranunculaceae | *Aconitum napellus* | Indicator | NA | NA | 1 | 1 | 17.65 |
| Ranunculaceae | *Adonis distorta* | Protected | NA | 92/43/CEE | 0 | 0 | 0.00 |
| Ranunculaceae | *Adonis pyrenaica* | Indicator | NA | NA | 0 | 0 | 0.00 |
| Ranunculaceae | *Anemone alpina* | Indicator | MD | NA | 0 | 0 | 0.00 |
| Ranunculaceae | *Anemone narcissiflora* | Indicator | NA | NA | 0 | 1 | 0.00 |
| Ranunculaceae | *Anemone nemorosa* | Indicator | NA | NA | 1 | 1 | 29.41 |
| Ranunculaceae | *Anemone patens* | Protected | MPD | 92/43/CEE | 1 | 1 | 5.88 |
| Ranunculaceae | *Anemone pavoniana* | Indicator | MPD | NA | 0 | 0 | 0.00 |
| Ranunculaceae | *Anemone scherfelii* | Indicator | NA | NA | 0 | 0 | 0.00 |
| Ranunculaceae | *Anemone vernalis* | Indicator | MD | NA | 1 | 1 | 23.53 |
| Ranunculaceae | *Aquilegia alpina* | Protected | NA | 92/43/CEE | 0 | 1 | 0.00 |
| Ranunculaceae | *Aquilegia pyrenaica* | Indicator | NA | NA | 0 | 0 | 0.00 |
| Ranunculaceae | *Caltha palustris* | Indicator | NA | NA | 1 | 1 | 35.29 |
| Ranunculaceae | *Helleborus foetidus* | Indicator | NA | NA | 1 | 1 | 17.65 |
| Ranunculaceae | *Helleborus niger* | Indicator | MPD | NA | 1 | 1 | 5.88 |
| Ranunculaceae | *Helleborus viridis* | Indicator | NA | NA | 0 | 0 | 0.00 |
| Ranunculaceae | *Hepatica nobilis* | Indicator | MPD | NA | 1 | 1 | 11.76 |
| Ranunculaceae | *Ranunculus acris* | Indicator | NA | NA | 1 | 1 | 47.06 |
| Ranunculaceae | *Ranunculus alpestris* | Indicator | MPD | NA | 0 | 1 | 0.00 |
| Ranunculaceae | *Ranunculus amplexicaulis* | Indicator | NA | NA | 0 | 0 | 0.00 |
| Ranunculaceae | *Ranunculus apenninus* | Indicator | NA | NA | 0 | 0 | 0.00 |
| Ranunculaceae | *Ranunculus bulbosus* | Indicator | ND | NA | 1 | 1 | 35.29 |
| Ranunculaceae | *Ranunculus carinthiacus* | Indicator | MPD | NA | 0 | 0 | 0.00 |
| Ranunculaceae | *Ranunculus gouanii* | Indicator | NA | NA | 0 | 0 | 0.00 |
| Ranunculaceae | *Ranunculus lanuginosus* | Indicator | NA | NA | 1 | 0 | 17.65 |
| Ranunculaceae | *Ranunculus montanus* | Indicator | NA | NA | 0 | 0 | 0.00 |
| Ranunculaceae | *Ranunculus oreophilus* | Indicator | NA | NA | 0 | 0 | 0.00 |
| Ranunculaceae | *Ranunculus parnassifolius* | Indicator | NA | NA | 0 | 0 | 0.00 |
| Ranunculaceae | *Ranunculus pollinensis* | Indicator | NA | NA | 0 | 0 | 0.00 |
| Ranunculaceae | *Ranunculus polyanthemos* | Indicator | NA | NA | 1 | 0 | 5.88 |
| Ranunculaceae | *Ranunculus pseudomontanus* | Indicator | NA | NA | 0 | 0 | 0.00 |
| Ranunculaceae | *Ranunculus pyrenaeus* | Indicator | NA | NA | 0 | 0 | 0.00 |
| Ranunculaceae | *Ranunculus repens* | Indicator | MPD | NA | 1 | 1 | 17.65 |
| Ranunculaceae | *Ranunculus ruscinonensis* | Indicator | NA | NA | 0 | 0 | 0.00 |
| Ranunculaceae | *Ranunculus serpens* | Indicator | NA | NA | 1 | 0 | 5.88 |
| Ranunculaceae | *Ranunculus thora* | Indicator | NA | NA | 0 | 0 | 0.00 |
| Ranunculaceae | *Ranunculus verna* | Indicator | NA | NA | 0 | 0 | 0.00 |
| Ranunculaceae | *Ranunculus villarsii* | Indicator | NA | NA | 0 | 0 | 0.00 |
| Ranunculaceae | *Thalictrum alpinum* | Indicator | MPD | NA | 0 | 1 | 0.00 |
| Ranunculaceae | *Thalictrum aquilegiifolium* | Indicator | NA | NA | 1 | 0 | 11.76 |
| Ranunculaceae | *Thalictrum minus* | Indicator | MPD | NA | 1 | 1 | 5.88 |
| Ranunculaceae | *Trollius europaeus* | Indicator | MPD | NA | 1 | 1 | 23.53 |
| Resedaceae | *Reseda decursiva* | Protected | NA | 92/43/CEE | 0 | 1 | 0.00 |
| Resedaceae | *Reseda lutea* | Indicator | PD | NA | 1 | 1 | 52.94 |
| Rosaceae | *Agrimonia eupatoria* | Indicator | PY | NA | 1 | 1 | 52.94 |
| Rosaceae | *Alchemilla alpigena* | Indicator | PD | NA | 0 | 0 | 0.00 |
| Rosaceae | *Alchemilla alpina* | Indicator | PD | NA | 1 | 1 | 11.76 |
| Rosaceae | *Alchemilla colorata* | Indicator | NA | NA | 0 | 0 | 0.00 |
| Rosaceae | *Alchemilla conjuncta* | Indicator | NA | NA | 0 | 0 | 0.00 |
| Rosaceae | *Alchemilla filicaulis* | Indicator | NA | NA | 0 | 0 | 0.00 |
| Rosaceae | *Alchemilla flabellata* | Indicator | NA | NA | 0 | 0 | 0.00 |
| Rosaceae | *Alchemilla glabra* | Indicator | NA | NA | 1 | 1 | 11.76 |
| Rosaceae | *Alchemilla hoppeana* | Indicator | NA | NA | 0 | 0 | 0.00 |
| Rosaceae | *Alchemilla hybrida* | Indicator | NA | NA | 0 | 0 | 0.00 |
| Rosaceae | *Alchemilla monticola* | Indicator | NA | NA | 1 | 1 | 11.76 |
| Rosaceae | *Alchemilla xanthochlora* | Indicator | NA | NA | 1 | 1 | 17.65 |
| Rosaceae | *Dryas octopetala* | Indicator | PD | NA | 0 | 1 | 0.00 |
| Rosaceae | *Drymocallis rupestris* | Indicator | NA | NA | 1 | 1 | 5.88 |
| Rosaceae | *Filipendula ulmaria* | Indicator | PD | NA | 1 | 1 | 58.82 |
| Rosaceae | *Filipendula vulgaris* | Indicator | NA | NA | 1 | 1 | 41.18 |
| Rosaceae | *Fragaria vesca* | Indicator | NA | NA | 1 | 1 | 52.94 |
| Rosaceae | *Fragaria viridis* | Indicator | NA | NA | 1 | 0 | 5.88 |
| Rosaceae | *Geum montanum* | Indicator | PD | NA | 1 | 1 | 5.88 |
| Rosaceae | *Geum pyrenaicum* | Indicator | PY | NA | 0 | 1 | 0.00 |
| Rosaceae | *Geum rivale* | Indicator | PD | NA | 1 | 1 | 47.06 |
| Rosaceae | *Geum sylvaticum* | Indicator | NA | NA | 0 | 0 | 0.00 |
| Rosaceae | *Geum urbanum* | Indicator | PD | NA | 1 | 1 | 41.18 |
| Rosaceae | *Potentilla alchimilloides* | Indicator | NA | NA | 0 | 0 | 0.00 |
| Rosaceae | *Potentilla aurea* | Indicator | PD | NA | 1 | 1 | 5.88 |
| Rosaceae | *Potentilla crantzii* | Indicator | ND | NA | 0 | 1 | 0.00 |
| Rosaceae | *Potentilla delphinensis* | Protected | NA | 92/43/CEE | 0 | 0 | 0.00 |
| Rosaceae | *Potentilla erecta* | Indicator | NA | NA | 1 | 1 | 23.53 |
| Rosaceae | *Potentilla glaucescens* | Indicator | NA | NA | 0 | 0 | 0.00 |
| Rosaceae | *Potentilla grandiflora* | Indicator | NA | NA | 0 | 0 | 0.00 |
| Rosaceae | *Potentilla humifusa* | Indicator | NA | NA | 0 | 0 | 0.00 |
| Rosaceae | *Potentilla montana* | Indicator | NA | NA | 0 | 0 | 0.00 |
| Rosaceae | *Potentilla nivea* | Indicator | ND | NA | 0 | 0 | 0.00 |
| Rosaceae | *Potentilla pusilla* | Indicator | NA | NA | 0 | 0 | 0.00 |
| Rosaceae | *Potentilla reptans* | Indicator | NA | NA | 1 | 1 | 11.76 |
| Rosaceae | *Potentilla rigoana* | Indicator | NA | NA | 0 | 0 | 0.00 |
| Rosaceae | *Potentilla tabernaemontani* | Indicator | NA | NA | 1 | 0 | 11.76 |
| Rosaceae | *Potentilla thuringiaca* | Indicator | NA | NA | 1 | 0 | 5.88 |
| Rosaceae | *Rosa pendulina* | Indicator | NA | NA | 1 | 0 | 5.88 |
| Rosaceae | *Rosa spinosissima* | Indicator | NA | NA | 1 | 0 | 5.88 |
| Rosaceae | *Rubus caesius* | Indicator | NA | NA | 0 | 1 | 0.00 |
| Rosaceae | *Rubus idaeus* | Indicator | PD | NA | 0 | 1 | 0.00 |
| Rosaceae | *Sanguisorba minor* | Indicator | ND | NA | 1 | 1 | 52.94 |
| Rosaceae | *Sanguisorba officinalis* | Indicator | PD | NA | 1 | 1 | 41.18 |
| Rosaceae | *Sibbaldia procumbens* | Indicator | PD | NA | 0 | 1 | 0.00 |
| Rubiaceae | *Asperula aristata* | Indicator | NA | NA | 0 | 0 | 0.00 |
| Rubiaceae | *Asperula cynanchica* | Indicator | NA | NA | 1 | 1 | 17.65 |
| Rubiaceae | *Asperula purpurea* | Indicator | NA | NA | 0 | 0 | 0.00 |
| Rubiaceae | *Cruciata glabra* | Indicator | NA | NA | 1 | 0 | 5.88 |
| Rubiaceae | *Cruciata laevipes* | Indicator | NA | NA | 1 | 1 | 11.76 |
| Rubiaceae | *Galium album* | Indicator | NA | NA | 1 | 0 | 5.88 |
| Rubiaceae | *Galium anisophyllon* | Indicator | NA | NA | 0 | 0 | 0.00 |
| Rubiaceae | *Galium estebanii* | Indicator | NA | NA | 0 | 0 | 0.00 |
| Rubiaceae | *Galium lucidum* | Indicator | NA | NA | 0 | 0 | 0.00 |
| Rubiaceae | *Galium magellense* | Indicator | NA | NA | 0 | 0 | 0.00 |
| Rubiaceae | *Galium marchandii* | Indicator | NA | NA | 0 | 0 | 0.00 |
| Rubiaceae | *Galium mollugo* | Indicator | NA | NA | 1 | 1 | 17.65 |
| Rubiaceae | *Galium noricum* | Indicator | NA | NA | 0 | 0 | 0.00 |
| Rubiaceae | *Galium obliquum* | Indicator | NA | NA | 0 | 0 | 0.00 |
| Rubiaceae | *Galium pumilum* | Indicator | NA | NA | 1 | 0 | 5.88 |
| Rubiaceae | *Galium pyrenaicum* | Indicator | NA | NA | 0 | 0 | 0.00 |
| Rubiaceae | *Galium saxatile* | Indicator | PD | NA | 1 | 1 | 5.88 |
| Rubiaceae | *Galium uliginosum* | Indicator | NA | NA | 1 | 1 | 11.76 |
| Rubiaceae | *Galium verum* | Indicator | PD | NA | 1 | 1 | 52.94 |
| Rubiaceae | *Galium viridiflorum* | Protected | NA | 92/43/CEE | 0 | 0 | 0.00 |
| Rutaceae | *Dictamnus albus* | Indicator | NA | NA | 1 | 1 | 23.53 |
| Salicaceae | *Salix alpina* | Indicator | NA | NA | 0 | 0 | 0.00 |
| Salicaceae | *Salix herbacea* | Indicator | ND | NA | 0 | 1 | 0.00 |
| Salicaceae | *Salix pyrenaica* | Indicator | NA | NA | 0 | 0 | 0.00 |
| Salicaceae | *Salix reticulata* | Indicator | PD | NA | 0 | 1 | 0.00 |
| Salicaceae | *Salix retusa* | Indicator | ND | NA | 0 | 1 | 0.00 |
| Salicaceae | *Salix serpyllifolia* | Indicator | NA | NA | 0 | 0 | 0.00 |
| Santalaceae | *Thesium ebracteatum* | Protected | NA | 92/43/CEE | 0 | 0 | 0.00 |
| Santalaceae | *Thesium linophyllon* | Indicator | NA | NA | 0 | 0 | 0.00 |
| Santalaceae | *Thesium pyrenaicum* | Indicator | NA | NA | 0 | 0 | 0.00 |
| Saxifragaceae | *Parnassia palustris* | Indicator | PD | NA | 1 | 1 | 11.76 |
| Saxifragaceae | *Saxifraga aizoides* | Indicator | PD | NA | 0 | 1 | 0.00 |
| Saxifragaceae | *Saxifraga bryoides* | Indicator | ND | NA | 0 | 0 | 0.00 |
| Saxifragaceae | *Saxifraga caesia* | Indicator | PD | NA | 0 | 1 | 0.00 |
| Saxifragaceae | *Saxifraga callosa* | Indicator | NA | NA | 0 | 0 | 0.00 |
| Saxifragaceae | *Saxifraga exarata* | Indicator | ND | NA | 0 | 0 | 0.00 |
| Saxifragaceae | *Saxifraga exarata moschata* | Indicator | ND | NA | 0 | 1 | 0.00 |
| Saxifragaceae | *Saxifraga granulata* | Indicator | NA | NA | 1 | 1 | 35.29 |
| Saxifragaceae | *Saxifraga hirculus* | Protected | NA | 92/43/CEE | 0 | 0 | 0.00 |
| Saxifragaceae | *Saxifraga hirsuta* | Indicator | NA | NA | 0 | 0 | 0.00 |
| Saxifragaceae | *Saxifraga hypnoides* | Indicator | ND | NA | 0 | 1 | 0.00 |
| Saxifragaceae | *Saxifraga oppositifolia* | Indicator | PD | NA | 0 | 1 | 0.00 |
| Saxifragaceae | *Saxifraga paniculata* | Indicator | PD | NA | 1 | 1 | 5.88 |
| Saxifragaceae | *Saxifraga stellaris* | Indicator | ND | NA | 0 | 1 | 0.00 |
| Saxifragaceae | *Saxifraga trifurcata* | Indicator | NA | NA | 0 | 0 | 0.00 |
| Saxifragaceae | *Saxifraga umbrosa* | Indicator | NA | NA | 0 | 0 | 0.00 |
| Saxifragaceae | *Saxifraga valdensis* | Protected | NA | 92/43/CEE | 0 | 0 | 0.00 |
| Scrophulariaceae | *Scrophularia canina* | Indicator | NA | NA | 0 | 0 | 0.00 |
| Scrophulariaceae | *Verbascum litigiosum* | Protected | NA | 92/43/CEE | 0 | 0 | 0.00 |
| Scrophulariaceae | *Verbascum longifolium* | Indicator | NA | NA | 0 | 1 | 0.00 |
| Scrophulariaceae | *Verbascum pulverulentum* | Indicator | NA | NA | 1 | 1 | 17.65 |
| Solanaceae | *Atropa baetica* | Protected | NA | 92/43/CEE | 0 | 0 | 0.00 |
| Solanaceae | *Mandragora officinarum* | Protected | NA | 92/43/CEE | 0 | 0 | 0.00 |
| Thymelaeaceae | *Daphne cneorum* | Indicator | NA | NA | 1 | 0 | 5.88 |
| Thymelaeaceae | *Daphne mezereum* | Indicator | PD | NA | 1 | 1 | 5.88 |
| Thymelaeaceae | *Daphne striata* | Indicator | PD | NA | 0 | 1 | 0.00 |
| Thymelaeaceae | *Thymelaea broteriana* | Protected | NA | 92/43/CEE | 0 | 0 | 0.00 |
| Tofieldiaceae | *Tofieldia calyculata* | Indicator | MPD | NA | 1 | 1 | 5.88 |
| Urticaceae | *Urtica dioica* | Indicator | PD | NA | 1 | 1 | 41.18 |
| Violaceae | *Viola biflora* | Indicator | PD | NA | 0 | 1 | 0.00 |
| Violaceae | *Viola calcarata* | Indicator | PD | NA | 0 | 1 | 0.00 |
| Violaceae | *Viola canina* | Indicator | NA | NA | 0 | 1 | 0.00 |
| Violaceae | *Viola cazorlensis* | Protected | NA | 92/43/CEE | 0 | 0 | 0.00 |
| Violaceae | *Viola cornuta* | Indicator | NA | NA | 0 | 0 | 0.00 |
| Violaceae | *Viola eugeniae* | Indicator | NA | NA | 0 | 0 | 0.00 |
| Violaceae | *Viola hirta* | Indicator | NA | NA | 1 | 1 | 5.88 |
| Violaceae | *Viola jaubertiana* | Protected | NA | 92/43/CEE | 0 | 0 | 0.00 |
| Violaceae | *Viola lactea* | Indicator | NA | NA | 0 | 1 | 0.00 |
| Violaceae | *Viola lutea* | Indicator | PD | NA | 1 | 1 | 5.88 |
| Violaceae | *Viola palustris* | Indicator | PD | NA | 1 | 1 | 5.88 |
| Violaceae | *Viola reichenbachiana* | Indicator | NA | NA | 0 | 1 | 0.00 |
| Violaceae | *Viola riviniana* | Indicator | PD | NA | 1 | 1 | 23.53 |
| Violaceae | *Viola rupestris* | Indicator | NA | NA | 0 | 0 | 0.00 |
| Violaceae | *Viola tricolor* | Indicator | NA | NA | 1 | 1 | 23.53 |
| Violaceae | *Viola tricolor ssp alpina* | Indicator | NA | NA | 0 | 0 | 0.00 |
| Xanthorrhoeaceae | *Asphodelus albus* | Indicator | NA | NA | 1 | 1 | 11.76 |

**Table S3:** 17 Seed suppliers across 17 countries used for data collection.

| Supplier | Supplier Name | Country | Total Species Commercially Available | Target Species Commercially Available |
| --- | --- | --- | --- | --- |
| Supplier A | Ottenberg | Austria | 149 | 70 |
| Supplier B | Ecosem | Belgium | 85 | 47 |
| Supplier C | Planta Naturalis | Czech Republic | 1058 | 252 |
| Supplier D | Nykilde | Denmark | 26 | 15 |
| Supplier E | Wildflowershop.co.uk | England | 239 | 118 |
| Supplier F | Semences du Puy | France | 84 | 40 |
| Supplier G | Rieger-Hofmann GmbH | Germany | 290 | 144 |
| Supplier H | Pannon Flora | Hungary | 345 | 123 |
| Supplier I | Design by Nature | R. of Ireland | 108 | 54 |
| Supplier J | Semenostrum | Italy | 98 | 53 |
| Supplier K | Bioforsk | Norway | 8 | 5 |
| Supplier L | Sementes de Portugal | Portugal | 306 | 35 |
| Supplier M | Agrosem Impex | Romania | 59 | 9 |
| Supplier N | Scotia Seeds | Scotland | 185 | 108 |
| Supplier O | Semillas Silvestres | Spain | 794 | 105 |
| Supplier P | Pratensis AB | Sweden | 82 | 55 |
| Supplier Q | Wildblumenburri Samen, Sprossen, Pflanzen | Switzerland | 376 | 186 |

**Table S4:** Statistics representing differences between variables in the percentage of suppliers with seed of each species commercially available compared across species groups (Figure S1). Kruskal-Wallis χ^2^ test and post-hoc pairwise Tukey and Kramer (Nemenyi) χ^2^ test, p-value statistics, indicating significance between group variables. Germination Data Available = ‘+ GDA’ , Germination Data Not Available= ‘- GDA ‘.

| Proportion of Producers with Commercial Availability | | | | | | | |  |
| --- | --- | --- | --- | --- | --- | --- | --- | --- |
| Kruskal-Wallis test across all species groups | | Chi-squared | | DF | | P-Value | |  |
|  | | 338.81 | | 5 | | < .001 | |  |
| Kruskal-Wallis post-hoc pairwise test | | | | | | | | |
| Species groups | Protected -GDA | Protected + GDA | Indicator - GDA | | Indicator + GDA | | Fodder - GDA | |
| Protected + GDA | 0.98 | - | - | | - | | - | |
| Indicator - GDA | 0.19 | 0.99 | - | | - | | - | |
| Indicator + GDA | <0 .001 | 0.005 | <0.001 | | - | | - | |
| Fodder - GDA | 0.92 | 0.99 | 1 | | 0.01 | | - | |
| Fodder + GDA | <0.001 | <0.001 | <0.001 | | 0.53 | | <0.002 | |

**Table S5:** The complete dataset summarised by Taxonomic family, in descending order of percentage of Commercial Availability (CA). #= number, %= percentage, Sp.= species, CA= Commercially Availability, GDA= Germination Data Availability

| Family | # of Sp. in Family | # of Sp. in Family with CA | % with CA | # of Sp. in Family with GDA | % with GDA |
| --- | --- | --- | --- | --- | --- |
| Xanthorrhoeaceae | 1 | 1 | 100 | 1 | 100 |
| Urticaceae | 1 | 1 | 100 | 1 | 100 |
| Tofieldiaceae | 1 | 1 | 100 | 1 | 100 |
| Rutaceae | 1 | 1 | 100 | 1 | 100 |
| Papaveraceae | 1 | 1 | 100 | 1 | 100 |
| Cupressaceae | 1 | 1 | 100 | 1 | 100 |
| Convolvulaceae | 1 | 1 | 100 | 1 | 100 |
| Apocynaceae | 1 | 1 | 100 | 1 | 100 |
| Amaranthaceae | 2 | 2 | 100 | 2 | 100 |
| Malvaceae | 3 | 2 | 66.67 | 0 | 0 |
| Polygonaceae | 10 | 6 | 60 | 7 | 70 |
| Lamiaceae | 43 | 24 | 55.81 | 23 | 53.49 |
| Geraniaceae | 9 | 5 | 55.56 | 5 | 55.56 |
| Resedaceae | 2 | 1 | 50 | 2 | 100 |
| Onagraceae | 2 | 1 | 50 | 2 | 100 |
| Linaceae | 8 | 4 | 50 | 5 | 62.50 |
| Thymelaeaceae | 4 | 2 | 50 | 2 | 50 |
| Hypericaceae | 8 | 4 | 50 | 4 | 50 |
| Crassulaceae | 12 | 6 | 50 | 5 | 41.67 |
| Rosaceae | 45 | 22 | 48.89 | 23 | 51.11 |
| Caprifoliaceae | 15 | 7 | 46.67 | 7 | 46.67 |
| Poaceae | 125 | 57 | 45.60 | 68 | 54.40 |
| Boraginaceae | 11 | 5 | 45.45 | 7 | 63.64 |
| Rubiaceae | 20 | 9 | 45 | 6 | 30 |
| Polygalaceae | 9 | 4 | 44.44 | 2 | 22.22 |
| Leguminosae | 97 | 43 | 44.33 | 62 | 63.92 |
| Compositae | 146 | 64 | 43.84 | 58 | 39.73 |
| Plantaginaceae | 39 | 17 | 43.59 | 23 | 58.97 |
| Ranunculaceae | 44 | 19 | 43.18 | 18 | 40.91 |
| Caryophyllaceae | 59 | 25 | 42.37 | 36 | 61.02 |
| Apiaceae | 53 | 22 | 41.51 | 25 | 47.17 |
| Euphorbiaceae | 5 | 2 | 40 | 1 | 20 |
| Campanulaceae | 31 | 12 | 38.71 | 9 | 29.03 |
| Amaryllidaceae | 17 | 6 | 35.29 | 3 | 17.65 |
| Juncaceae | 15 | 5 | 33.33 | 10 | 66.67 |
| Violaceae | 16 | 5 | 31.25 | 10 | 62.50 |
| Cyperaceae | 40 | 12 | 30 | 26 | 65 |
| Brassicaceae | 30 | 9 | 30 | 16 | 53.33 |
| Liliaceae | 7 | 2 | 28.57 | 2 | 28.57 |
| Asparagaceae | 14 | 4 | 28.57 | 4 | 28.57 |
| Primulaceae | 19 | 5 | 26.32 | 8 | 42.11 |
| Scrophulariaceae | 4 | 1 | 25 | 2 | 50 |
| Ericaceae | 10 | 2 | 20 | 6 | 60 |
| Colchicaceae | 5 | 1 | 20 | 2 | 40 |
| Saxifragaceae | 17 | 3 | 17.65 | 9 | 52.94 |
| Cistaceae | 6 | 1 | 16.67 | 3 | 50 |
| Orobanchaceae | 33 | 5 | 15.15 | 17 | 51.52 |
| Plumbaginaceae | 10 | 1 | 10 | 2 | 20 |
| Gentianaceae | 25 | 2 | 8 | 17 | 68 |
| Orchidaceae | 17 | 1 | 5.88 | 4 | 23.53 |
| Oxalidaceae | 1 | 0 | 0 | 1 | 100 |
| Salicaceae | 6 | 0 | 0 | 3 | 50 |
| Iridaceae | 6 | 0 | 0 | 1 | 16.67 |
| Solanaceae | 2 | 0 | 0 | 0 | 0 |
| Santalaceae | 3 | 0 | 0 | 0 | 0 |
| Paeoniaceae | 2 | 0 | 0 | 0 | 0 |
| Melanthiaceae | 2 | 0 | 0 | 0 | 0 |
| Lentibulariaceae | 2 | 0 | 0 | 0 | 0 |
| Grossulariaceae | 1 | 0 | 0 | 0 | 0 |
| Cruciferae | 3 | 0 | 0 | 0 | 0 |

**
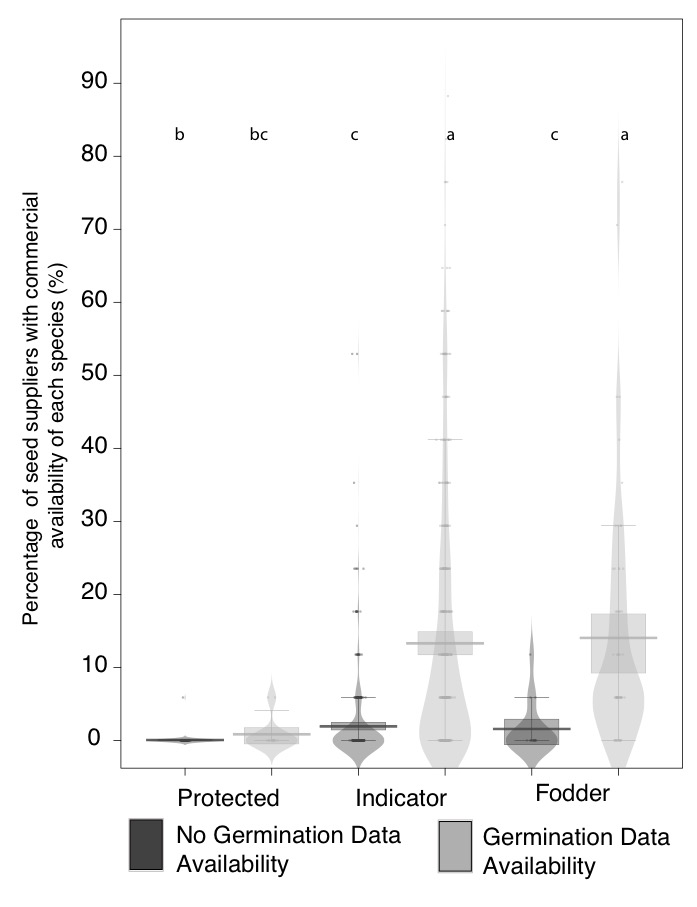
**

**Figure S1:** Observed percentage (%) of producers (total 17) with *commercial availability* of seed with and without *germination data availability* in each species group. RDI plots (Raw data, Descriptive and Inference statistics) show jittered points of raw data, centre bars indicate the mean of the data, beans outline the smoothed density of the data, whiskers mark the 10% and 90% quantiles of the data, and inference bands show the Bayesian 95% High Density Interval inferential statistics for each group. Letters show statistical differences between groups (Table S4).
